# Examining Event-Related Potential (ERP) Correlates of Decision Bias in Recognition Memory Judgments

**DOI:** 10.1371/journal.pone.0106411

**Published:** 2014-09-29

**Authors:** Holger Hill, Sabine Windmann

**Affiliations:** 1 Goethe University Frankfurt, Institute for Psychology, Cognitive Psychology II, Frankfurt, Germany; 2 Karlsruhe Institute for Technology (KIT), Institute of Sports and Sports Sciences, Department of Applied Psychology, Karlsruhe, Germany; Vanderbilt University, United States of America

## Abstract

Memory judgments can be based on accurate memory information or on decision bias (the tendency to report that an event is part of episodic memory when one is in fact unsure). Event related potentials (ERP) correlates are important research tools for elucidating the dynamics underlying memory judgments but so far have been established only for investigations of accurate old/new discrimination. To identify the ERP correlates of bias, and observe how these interact with ERP correlates of memory, we conducted three experiments that manipulated decision bias within participants via instructions during recognition memory tests while their ERPs were recorded. In Experiment 1, the bias manipulation was performed between blocks of trials (automatized bias) and compared to trial-by-trial shifts of bias in accord with an external cue (flexibly controlled bias). In Experiment 2, the bias manipulation was performed at two different levels of accurate old/new discrimination as the memory strength of old (studied) items was varied. In Experiment 3, the bias manipulation was added to another, bottom-up driven manipulation of bias induced via familiarity. In the first two Experiments, and in the low familiarity condition of Experiment 3, we found evidence of an early frontocentral ERP component at 320 ms poststimulus (the FN320) that was sensitive to the manipulation of bias via instruction, with more negative amplitudes indexing more liberal bias. By contrast, later during the trial (500–700 ms poststimulus), bias effects interacted with old/new effects across all three experiments. Results suggest that the decision criterion is typically activated early during recognition memory trials, and is integrated with retrieved memory signals and task-specific processing demands later during the trial. More generally, the findings demonstrate how ERPs can help to specify the dynamics of recognition memory processes under top-down and bottom-up controlled retrieval conditions.

## Introduction

Reports about past events and experiences not only rely on accurate retrieval of stored information in memory, but also on subjective decision-making processes, also called response biases. The latter play a role especially when the memory trace is weak or retrieval is difficult so that individuals are unsure, but need to report nonetheless as in forced choice situations. Signal-detection theory and nonparametric decision models [1]–[6] have provided memory researchers with mathematical tools to statistically separate accurate memory (sensitivity) from decision bias by analyzing behavioral response rates observed in laboratory experiments.

A growing number of experimental studies have shown that variation in memory decision bias is not merely a nuisance variable that needs to be controlled for, but may itself contain important information about cognitive states, processes, and even traits [7]–[9]. For instance, shifts in decision bias have been shown to vary in accord with participants' goal motivations and emotions [10]–[13]. Seemingly paradoxically, even illusory memories can be reflected in measures of decision bias [14], [15].

### Separating memory from decision bias acccording to behavioral models

**Figure 1:**
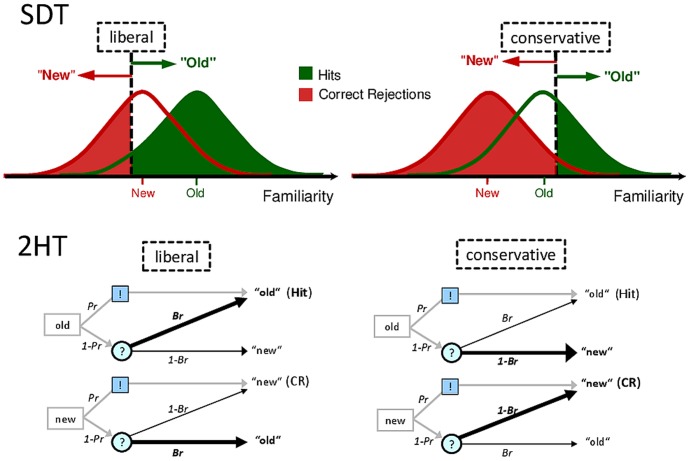
In Signal Detection Theory (SDT), studied (old) items elicit a higher feeling of familiarity relative to unstudied (new) items, both with a normal distribution. The mean familiarity difference between the old and new item distributions reflects recognition memory (old/new discrimination performance, or d′). By contrast, the point on the familiarity dimension above which “old” responses are rendered and below which “new” responses are rendered reflects the decision criterion (or response bias, c). More items with relatively low familiarity are contained in the Hit response category (blue area) when the threshold is liberal as compared to conservative; the same is true for Correct Rejections (red area). In the **Two High Threshold Model (2 HTM)**, Hit Rates (*HR*) reflect the probability that an old item is either correctly recognized (*Pr*), or not (1 - *Pr*) while there is a bias to respond “old” (*Br*). This is mathematically formulated as: [1] *HR* = *Pr*+(1 - *Pr*) *Br*. On the other hand, False Alarm Rates (*FAR*) reflect the probability that a new item is not recognized (1 - *Pr*) while there is a bias to respond “old” (*Br*), mathematically formulated as: [2] *FAR* = (1 - *Pr*) *Br*. Using [2] to resolve *Pr* in [1] yields: *HR* = *Pr*+*FAR*, equivalent to *Pr* = *HR – FAR.* Rewriting [2] yields *Br* = *FAR/(1 - Pr)*. Since recognition thresholds for old and new items are assumed to be equal, *Pr* and *Br* can be computed analogously from Correct Rejection Rates and Miss Rates. Thick lines in the decision tree depict different response tendencies under conditions of relatively liberal (high *Br*) as opposed to conservative (low *Br*) decision criteria. **For both models**, SDT and 2 HT, if accurate old/new discrimination is equal, changes in Hits, Correct Rejections, or any response types, can only stem from bias, whether these are changes in response rates, confidence ratings, or associated brain measures.

Some of the findings on the functions of response bias depend on the appropriateness of assumptions made by the underlying statistical model, often leading to heated debates about the valid interpretation of the behavioral data [16]–[25]. Two classes of models for simple old/new recognition memory tasks are depicted in Figure 1. **Signal Detection Theory (SDT)**, presumes that items in a recognition memory test elicit a feeling of familiarity that corresponds with the item's position on a continuous memory strength dimension. The mean familiarity signal is higher for studied items than for unstudied items while being normally distributed for both item types due to random variation. This mean difference in familiarity between studied and unstudied items reflects accurate old/new recognition (or d′ for “discrimination”, [2]). However, to the degree that the two item distributions overlap, accurate old/new discrimination is not possible, so that participants need to guess depending on a decision threshold. This threshold (c for criterion, [2]) is defined as the point on the memory strength dimension above which participants respond “old” and below which they respond “new”. The decision criterion is presumed to be independent of old/new discrimination, but the two parameters are unreliable and can appear correlated when the two item distributions are not normal. Alternative estimates of bias and accuracy have therefore been proposed that do not make distributional assumptions [4], [26].

The second class of models consists of discrete state models, of which the most well-known variant, the **two-high threshold model (2 HTM)**, is depicted in Figure 1. The model postulates the following decision tree: When an old (studied) test item is presented, the item is correctly recognized (i.e., it surpasses the threshold for old recognition) with a probability of *Pr* (for “probability of recognition”, [26]), in which case a correct “old” response is given (hit). When a new item is presented, it is correctly recognized with the same probability *Pr* (surpassing the threshold for new item recognition), in which case a correct “new” response is rendered (correct rejection). However, if the old/new status of a test item is not recognized (probability (1-*Pr*)), the system is in a state of uncertainty and can only guess. With the (conditional) probability of response bias *Br*, an “old” response is rendered, and with the complementary (conditional) probability of (1 – *Br*), a “new” response is entered on the basis of guessing. Hence, *Br* quantifies the probability to guess “old” when recognition memory fails.

Some researchers have found the continuous SDT more appropriate than discrete state models [22], [27], while others find the opposite [21], [23], and yet others find agreement between the two [28]. To advance such theoretical discussions, it would be valuable to have an index of decision bias in memory research that is not statistically extracted from behavioral response rates, but more directly observed from the underlying brain processes. Event-related potentials (ERPs) are ideal candidates to that end for their sensitivity to cognitive processes and their high temporal resolution.

### Using ERPs to investigate decision bias in memory judgments

Researchers have long used ERPs to elucidate and analyze the various cognitive processes involved in recognition memory (as reviewed, e.g., by refs [29]–[33]). The typical and highly robust finding is that old (studied) test items elicit more positive going waveforms relative to new (unstudied) test items. Abundant research has documented these ERP old/new differences, but the effects of decision criteria (or the resulting response bias) on ERPs recorded during recognition memory tasks have been investigated quite rarely.

Two ERP investigations of response bias exist that focused on the effects of *emotions* on shifts of the criterion to respond “old” in an old/new recognition memory task [34], [35]. These studies identified a reduced ERP old/new effect at prefrontal sites between 300 and 500 ms as the correlate of a more liberal decision-criterion that was associated with emotionally negative items relative to emotionally neutral items. The same correlate was later found for a recognition memory task involving only neutral words in comparing individuals generally adopting a liberal bias with those adopting a conservative bias [36]. Importantly, in that study, the overall bias (averaged across old/new conditions) also correlated positively (.453) with the overall negative ERP amplitude recorded at prefrontal sites, an important piece of evidence that is independent of ERP old/new differences.

[37] confirmed and extended these findings by varying decision criteria within participants via instructions: In one condition, participants were asked to respond conservatively to old items (respond “old” only when sure that the item is old); in the other response condition, participants were asked to make a conservative decision to new items (i.e., reject only when sure that the item is new). Consistent with the prior studies, they found an early (300–500 ms poststimulus) old/new effect in a frontal ERP component presumably reflecting familiarity (as opposed to recollection) differentiating the two bias conditions. Furthermore, their data also showed more positive overall amplitudes in the conservative condition relative to liberal. Importantly, and grossly in line with SDTs assumption of statistical independence of bias and accuracy, in all these studies, there were no confounding differences in accurate memory performance between the bias conditions.

In summary, evidence shows that variations in the criteria underlying old/new decisions appear to affect relatively early ERP measures at frontal sites. As such, they precede consciously controlled memory processes and occur approximately coincidentally with unconscious, implicit memory retrieval and automatic familiarity effects [29]–[32]. The response bias thus has been construed to reflect the default setting, or response gate, for “old” decisions that is (relatively) wide open in the case of a liberal criterion, and (relatively) occluded in the case of a conservative criterion, and then awaits to be either confirmed or overturned by the later retrieved memory information [36].

Some researchers have interpreted the ERP correlates of decision criterion in terms of memory strength, indexing different degrees of familiarity required for “old” judgments [37], while others describe the criteria independent of item retrieval processes in terms of decision-making as part of executive control processes [34], [36]. The former interpretation appears to relate more to stimulus attributes (familiarity), with the bias becoming more liberal when both item distributions are shifted upwards on the familiarity dimension (Figure 1). By contrast, the latter account refers to criterion setting in terms of a top-down controlled process that depends on preferences and goal states of the individual. For the behavioral parameters derived from SDT and the 2 HTM, these two processes are indistinguishable, for both lead to changes in the bias measure, despite the different underlying cognitive processes. ERP correlates might help to differentiate these two mechanisms, but given the sparse empirical basis, and the lack of direct comparisons, it is at present unclear whether and exactly what ERP correlates of decision bias are sensitive to manipulations of either top-down or bottom-up driven processes indexing executive control or familiarity, respectively.

### The present study

In a series of three experiments, we pursued two goals. First, we set out to find ERP correlates of decision bias that are independent of memory task demands. That is, across three different variants of recognition memory tasks, we searched for a common denominator in the ERPs that would be sensitive and specific to bias effects. These effects would be reflected in ERP *main effects* of bias in all three memory tasks. Second, we investigated ERP correlates of bias that would depend on or vary with memory task demands, and would therefore be influenced by memory task context. These latter processes would be specific for the memory task performed, and would be reflected in *interactions* of ERP correlates of bias with memory processes.

In all experiments, participants performed recognition memory tasks with words while their decision bias was varied via instructions. In addition, one other variable was manipulated, setting the particular memory task context. Specifically, top-down (self-controlled, goal-related) as opposed to bottom-up driven processes (automatic, familiarity-related) were manipulated in either memory retrieval or decision-making:

In Experiment 1, we varied the degree of top-down executive control involved in criterion-setting in addition to the bias manipulation via instructions: In one condition (block), the bias was manipulated blockwise so that criterion-setting processes were allowed to automatize, whereas in the other condition (random), the bias manipulation occurred randomized across trials such that participants had to flexibly shift their decision criterion on a trial-by-trial basis depending on an external cue as in a task-switching paradigm. In essence, the task varied memory of (or retrieval of) criterion activation: automatized versus controlled.

In Experiment 2, automatic memory, more specifically, bottom-up driven familiarity, of studied items was varied in addition to a standard (blockwise) bias manipulation via instruction: Half of the old items were presented three times at study instead of only once to boost their familiarity, shifting the distribution of old items upwards on the memory strength dimension (thereby facilitating old/new discrimination). This design allowed us to compare ERP effects of bias at two different levels of accurate old/new recognition memory to test the independence assumption of SDT and 2 HTM.

Experiment 3 again manipulated bottom-up driven familiarity; however, not only in studied (old) items, but also unstudied (new) items, via a separate reading task presented before the study phase of the recognition memory task, with the intention to shift distributions of both old and new items upwards on the memory strength dimension. Again, this manipulation was performed in parallel to the same blockwise manipulation of bias as in Experiments 1 and 2. We presumed that the reading task would induce a retrieval bias for highly familiar items, analogously to the process dissociation procedure [38]. We expected this manipulation to affect the response bias and were interested in how this would affect the bias manipulation via instruction as reflected in the associated ERPs.

In summary, our experiments meant to determine and challenge the sensitivity and the consistency of ERP bias effects in different recognition memory task contexts. All experimental manipulations were performed within participants. Given the prior studies on ERP bias effects and their relation to familiarity [33], [37], we focused specifically on the time window of 300–500 ms poststimulus at frontocentral recording sites, consistent with familiarity effects obtained after averaged reference transformation, c.f. [23], [33]). Visual inspection of the grand averages revealed a frontocentral component around 320 ms poststimulus in all three Experiments that served as the ROI in the main statistical analyses. In addition, we analyzed standard ERP recognition memory measures by taking amplitudes from frontal [33]–[36] and parietal electrodes in early (300–500 ms poststimulus) and late (500–700 ms poststimulus) time-windows [39], [29], [36]). For descriptive purposes, T-maps of all effects between 200 and 800 ms poststimulus are shown for all electrode sites in the Supporting Information, Figures S1–S3.

Following common practice in ERP memory research, we used only correct response trials in the ERP analyses. This would maximize old/new differences and also make our results comparable with prior publications. Note that in this design, ERP correlates of accurate memory refer to differences between old and new items whereas ERP correlates of bias refer to differences between liberal and conservative response criteria. Both effects are obtained because different types of items are contained in the ERP averages: In the case of accurate old/new recognition, ERPs averaged across correctly recognized old items (hits) are compared with ERPs averaged across correctly recognized new items (correct rejections), with the resulting ERP difference reflecting the mean familiarity difference between old and new item distributions in terms of SDT (see Figure 1). In the case of bias, ERPs averaged across items falling above the conservative response criterion on the memory strength dimension are compared to ERPs averaged across items falling above the liberal response criterion. Both item types are judged “old”, but the latter type (liberal) contains more trials associated with relatively low levels of familiarity compared to the former (conservative). That same comparison of conservative versus liberal is then again performed on ERPs averaged across items falling below the response threshold (items judged “new”), and should yield the same ERP difference, if assumptions of SDT hold. In both cases, the conservative/liberal difference reflects the ERP correlate of two different thresholds on the familiarity dimension, thresholds above which “old” responses are rendered and below which “new” responses are rendered (Figure 1). For these comparisons, it is not relevant whether the compared items are correctly recognized (as in our case) or not, for as long as the comparison is performed between conservative and liberal response criteria.

Contrary to SDT, 2 HTM makes no assumptions on the degree of familiarity associated with a high versus low decision threshold (*Br*), in fact, the model makes no claim about the cognitive or representational dimension underlying different guessing biases. In that sense, taken at face value, 2 HTM appears closer to executive control accounts of response bias that describe decision criteria as top-down controlled in accordance with goals, expectations, and preferences rooted outside the memory domain, and not directly in terms of threshold points on the familiarity dimension. This is the main reason why we chose to use 2 HTM of bias and accuracy in our main analyses. Another commonality of the two frameworks is that the ERP correlates of accuracy and bias should be statistically independent of one another; in fact, Snodgrass and Corwin [26] found this assumption to be most valid for the 2 HTM. Hence, we expected ERP bias effects to show either a different topography or a different time course than ERP effects of accurate memory.

## Experiment 1

In Experiment 1, we varied the decision criterion by standard means through instruction in two ways: In one condition (block), participants were asked to use either a liberal or a conservative bias constantly across blocks of 160 recognition memory test trials; in the other condition (random), they were instructed to vary the decision criterion (liberal or conservative) in accord with a cue given directly prior to each memory test trial. The first condition allowed the bias to automatize within large blocks of trials, whereas the latter condition required executive control to flexibly modulate the bias on a trial-by-trial basis, as in a typical task-switching paradigm. The manipulation was meant to provoke dynamic adaptations of criterion-setting (or criterion-activation) processes, including temporal adjustments, owing to cognitive control processes induced by task context.

### Materials and Methods Experiment 1

#### Participants

Data were obtained from 34 participants (students) who participated for course credit or a monetary reward of 20 Euro. All participants were healthy native German speakers with normal or corrected-to-normal vision and gave signed informed consent before participating in the experiment. Data of two participants had to be excluded due to an insufficient quality of the ERP data.

Of the remaining 32 participants, 8 did not comply with task instructions in at least one of the two conditions (random or block). That is, these participants did not adopt a more liberal bias in the liberal condition relative to conservative. This happened apparently because some participants confused the conditions on some of the trials, especially directly after experimental blocks had changed. We excluded these non-conforming participants from the main analysis but show their performance and ERPs in Figure S4.

The final sample consisted of 24 participants (16 females). Mean age was 21.5 years (range 19–28). Handedness was assessed using a German version of the Edinburgh Inventory [40], twenty-three participants were right-handed.

#### Stimuli and stimulation sequences

Eight lists of 80 emotionally neutral words each were created using the German Handbook of Word Norms [41]. Lists were constructed to be parallel (and were in fact not significantly different) with regards to mean word length (numbers of syllables between 2–3, numbers of letters between 5–10), valence, arousal, concreteness, and word frequency (frequencies were obtained from the Celex database, Centre for Lexical Information, Max Planck Institute for Psycholinguistics, Nijmegen, The Netherlands). Lists were randomly assigned to the experimental conditions; lists of old and new words were counterbalanced across participants. For all conditions (random/block and conservative/liberal), the study list contained 40 words, and the recognition test list contained 80 words (40 old and 40 new).

#### Procedures

Stimuli were presented in the center of a computer screen using the Presentation 10.2 software (Neurobehavioral Systems, www.neurobs.com). Given a viewing distance of about 1 m, visual angle of the stimulation was up to 3° in width and 0.6° in height. For the study phase, words were displayed for 400 ms followed by an interstimulus interval (ISI) of 1400 ms. Participants were instructed to read these words attentively, so without any reference to item retention and later retrieval.

For the recognition phase in the block condition, participants were cued before each block of 40 test items about the decision criterion to use (liberal or conservative, quasi-randomized order) with either an image of a lady lying relaxed in a deckchair (liberal condition) or a sternly looking lady (conservative condition); the images were taken from the International Affective Picture System (IAPS, images 2037 and 2372) [42]. In the random condition, participants were instructed before each trial about the decision type to use (liberal, conservative) with the corresponding cue image shown for 500 ms, followed by a blank screen for 900–1100 ms (randomized duration). In the conservative condition, participants were instructed to “respond ‘old’ (using the mouse button) only when you know for sure that the word had been presented in the study phase, or otherwise press ‘new’”. In the liberal condition, they were instructed to respond ‘old’ when they had “a feeling that the word might have been presented in the study phase, or otherwise press ‘new’”. In both conditions, on each trial a fixation cross was presented for a randomly varying duration of 1400 to 1800 ms followed by the test word displayed for up to 3 sec or until the participant made a response. Participants were instructed to respond as fast and accurately as possible.

Participants obtained practice trials prior to testing. They then started either with all blocks of the random condition or with all blocks of the block condition, with liberal and conservative conditions balanced across participants. Breaks between blocks of 40 trials as well as between study and recognition test were self-paced. They were asked to blink or move only during intertrial intervals if possible.

#### EEG recordings

A 62-channel EEG was recorded continuously with BrainAmp DC-amplifiers (BrainProducts, Gilching, Germany; sample rate 250 Hz, resolution 0.1 µV/bit, input-impedance 10 MOhm) using an equidistant EasyCap (EasyCap GmbH, Herrsching-Breitbrunn, Germany, www.easycap.de) with sintered Ag/AgCl electrodes. Eye blinks and movements were monitored with supra- and infra-orbital electrodes and with electrodes on the external canthi. The vertex electrode was used as reference. To avoid injuries due to skin abrasion, electrode impedances were kept below 20 kOhm which is considered more than sufficient from the electrical engineering perspective [43], [44].

#### Data analysis

Accurate old/new recognition memory *Pr* = *HR - FAR* and the response bias *Br* = *FAR*/(1 - *Pr*) were computed according to 2 HTM [3], [26], where *HR* is the probability of “old” responses to old items, and *FAR* is the probability of “old” responses to new items. Correct rejections (*CR*) were defined as the probability of “new” responses to new items. We additionally computed nonparametric SDT parameters *A* for accuracy and *b* for bias following [4], and the parametric estimates *d′* and *c* following [2], with cases of *FAR* = 0 or *HR* = 1 set to missing (as opposed to corrected) as this occurred less than 2 times per condition.

The EEG was analyzed using the Vision Analyzer 1.05 software (BrainProducts, Gilching, Germany; www.brainproducts.com). EEG data were digitally filtered with 30 Hz/24 dB Butterworth zero phase lowpass and 0.1 Hz/12 dB highpass, segmented into epochs of −200 ms to 1500 ms around stimulus onset, and baseline corrected (−200 to 0 ms). After removing segments with very large artefacts (exceeding ±500 µV), eye blinks were corrected using independent component analysis (ICA). ICA components containing eye blink activity were identified by inspecting their topographical distribution, and comparing the time course of components and EEG for co-occurence of blinks in random samples. After removing the blink component(s), success of this procedure was controlled by comparing the EEG data from before and from after the correction. Furthermore, accuracy of ICA blink removal was checked by comparing the results of blink removal with elimination of blink trials. Because the study phase contained a total of 320 segments, a sufficient number of blink-free segments was available for this comparison. Data were baseline corrected again to remove offset inaccuracies due to ICA blink removal. After applying a semiautomatic procedure for artefact detection (amplitude criterion ±50 µV, gradient 20 µV/sample), the complete datasets were inspected again visually. Traces of single channels containing artefacts were removed. If there were more than ten contaminated traces, the whole segment was removed. Only participants were included with at least 15 segments in each condition, provided that standard visual evoked potentials (P100, N170) were clearly visible. Segments were averaged separately for correct response trials of the different experimental conditions. Segments with response times outside the time window of 100 ms to 1500 ms poststimulus were removed. Averages were rereferenced (average reference transform [45]), and the reconstructed vertex reference was added to the data, resulting in 61 EEG channels.

For the frontocentral negative component (FN320), ERP amplitudes were analyzed at FCz and the six surrounding electrodes (Fz, F1, F2, FC1, FC2, Cz) between 300 and 350 ms poststimulus. For the standard analyses, frontal ERP amplitudes were taken at Fz and the six surrounding electrodes (AFz, AF3, AF4, F1, F2, FCz) and parietal sites (Cz, CP1, CP2, CPz, P1, P2, Pz) in early (300–500 ms) and late (500–700 ms poststimulus) time windows, respectively. These amplitude measures were analyzed using ANOVAs with the four repeated measures factors Electrode Site, Block (block/random), Criterion (liberal/conservative), and Old/New. Greenhouse-Geisser corrected *p*-values were applied when needed. The Newman-Keuls test was used for post-hoc tests.

### Results Experiment 1

#### Behavioral data

**Figure 2:**
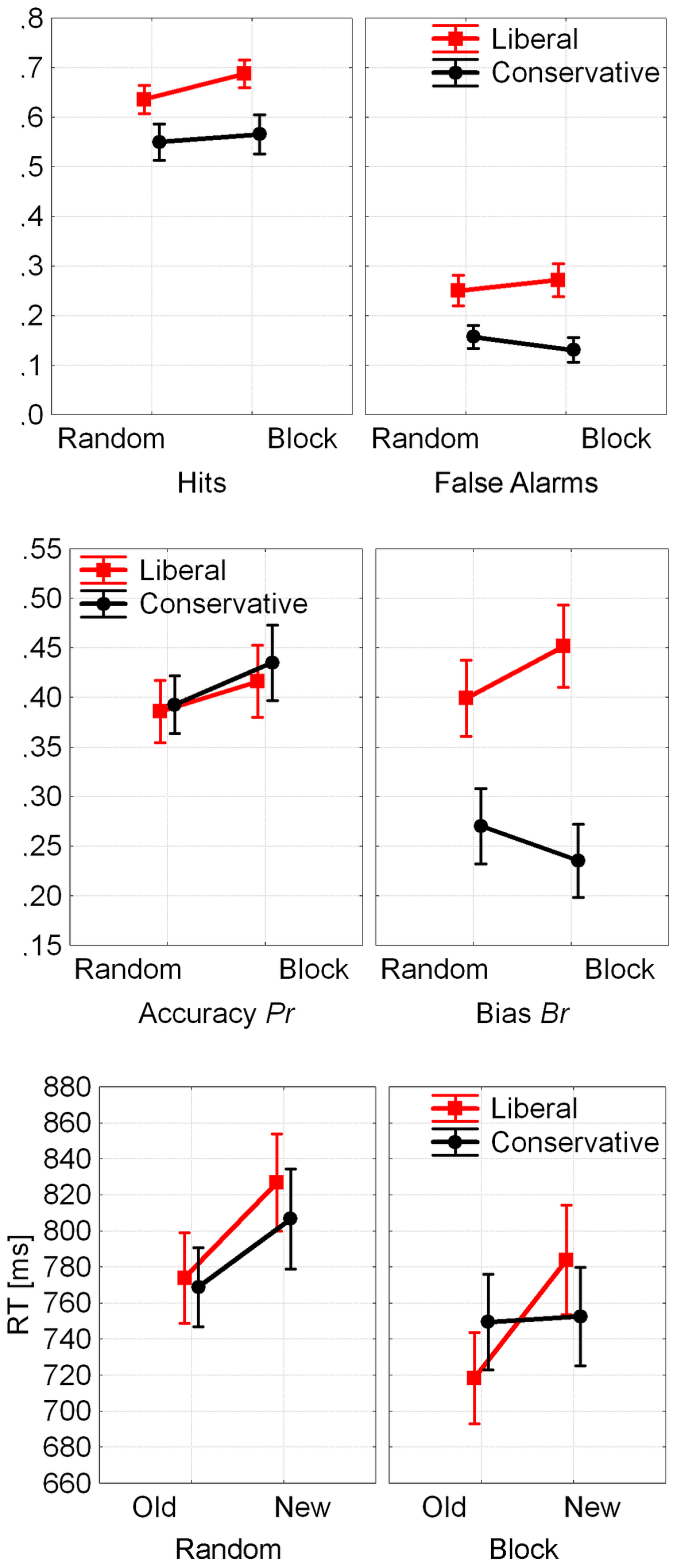
Hitrates (HR) and False Alarm Rates (FAR, top), Bias *Br* and Accurate Old/New Recognition *Pr* (center), and RTs (bottom) for Experiment 1 (error bars display standard errors).

Figure 2 displays hit rates, false alarm rates, bias, accuracy, and RTs in the four experimental conditions. The two-way (Block x Criterion) ANOVA of the bias measure *Br* revealed a main effect for criterion, as expected (conservative < liberal; *F*(1, 23) = 88.9, *p*<.001, *eta^2^* = 0.79), and a significant Block x Criterion interaction: *F*(1, 23) = 7.76, *p*<.011, *eta^2^* = 0.25, indicating a significantly smaller conservative < liberal bias difference in the random condition relative to block, in line with our presumption that shifting the bias in the random condition would be more difficult relative to block. Analysis of accurate old/new recognition memory *Pr* revealed no significant effects.

Parameters *Br* and *Pr* were highly correlated with the SDT indices on bias and accuracy, respectively (average correlations of *r* = .90), as detailed in Table S1 of the Supporting Information.

RTs for correct responses (Figure 2, bottom) were analyzed by an ANOVA with the three within-subjects factors Response Type (old/new), Criterion (liberal/conservative), and Block (block/random). The main effects revealed faster responses for old vs. new items: *F*(1, 23) = 8.95, *p* = .001, *eta^2^* = 0.28, as is typical for recognition memory judgments, as well as faster responses for the blocked vs. random condition: *F*(1, 23) = 4.59, *p*<.05, *eta^2^* = 0.17, again confirming that the block condition was easier than the random condition. The interaction Criterion x Old/New was also significant: *F*(1, 23) = 15.04, *p*<.001, *eta^2^* = 0.4. Furthermore, the three-way interaction was close to significance: *F*(1, 23) = 4.14, *p*<.054, *eta^2^* = 0.15. Figure 2 shows that the pattern results from the fact that speeded responses to old items as compared to new items were observed in all conditions except the conservative condition in the blocked trials, where this difference was much smaller and almost nonexistent.

#### ERP data

**Figure 3:**
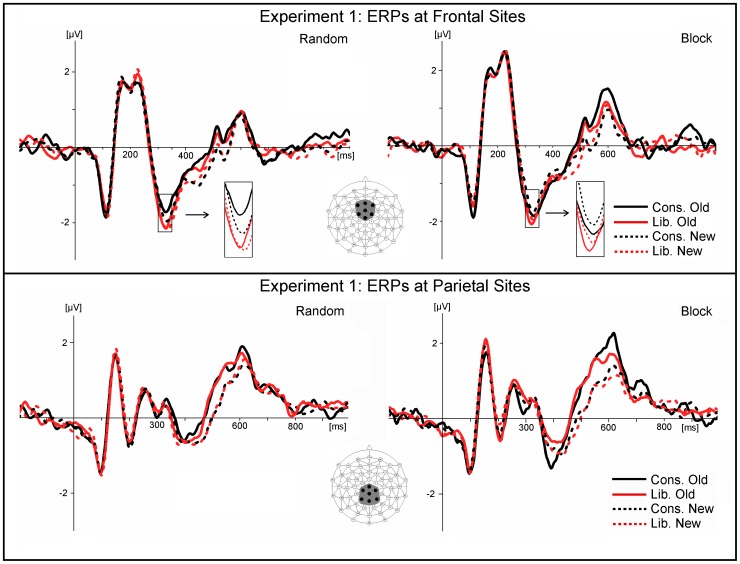
Only data of the 24 participants who varied the decision criterion in line with instructions are included in this Figure. TOP: Frontocentral grand average ERPs for the random condition (left) and the block condition (right) of Experiment 1. The negative component (FN320) peaking around 320 ms was larger (more negative) for the liberal than for the conservative decision criterion. BOTTOM: Parietal grand average ERPs for the random condition (left) and the block condition (right) of **Experiment 1**. The displayed waveforms are averaged across the seven sites included in the statistical analysis (see inserts).

The frontocentral negativity peaking around 320 ms with a maximum at FCz (FN320) was sensitive to the effects of criterion (Figure 3). The ANOVA of mean amplitudes (time window 300–350 ms) taken at FCz and the six surrounding sites (see insert in Figure 3) revealed that the component was larger (more negative) in the liberal compared to the conservative condition (*F*(1, 23) = 12.40, *p*<0.002, *eta^2^* = 0.35; Figure 3); a small potential difference that was highly consistent across participants. There were no further effects except for a main effect for Electrode Site: *F*(6, 138) = 8.38, *p*<.001, *eta^2^* = 0.27 indicating that the size of the bias effect varied somewhat within the cluster.

Notably, across all participants, including the eight participants that were excluded from the main analysis because they failed to shift their decision criteria in line with instructions (see Figure S4), bias effects in the FN320 correlated positively with variations in the behavioral measure *Br* (Pearson's *r* = .56, *N* = 32, *p*<.001). That is, the more the participants varied their decision criterion in line with instructions, the larger were the observed amplitude differences in FN320 (liberal higher FN320 amplitude than conservative). In the subsample of participants who did comply with instructions, the correlation was also positive albeit not significant (Pearson's *r* = .36, *N* = 24, *p*<.82).

Results from the standard analysis of ERP old/new effects are shown in Tables 1 and 2.

**Table 1:** Results of the ANOVA of **frontal** ERP amplitudes.

Experiment	Experiment 1		Experiment 2*		Experiment 3	
Design (Factor)	Random/Block		Condition (HighFam, LowFam, New)		Familiarity (High/Low)	
**Time Window (ms)**	**Early (300–500)**	**Late (500–700)**	**Early (300–500)**	**Late (500–700)**	**Early (300–500)**	**Late (500–700)**
Old/New			*F*(2, 48) = 16.02, *p*<.001, η_p_^2^ = .40 *Post hoc*: (HighFam = LowFam) > New		*F*(1, 25) = 23.47, *p*<.001, η_p_^2^ = 48	*F*(1, 25) = 7.49, *p* = .011, η_p_^2^ = .23
Criterion (Lib/Con)	*F*(1, 23) = 8.78, *p* = .007, η_p_^2^ = .28		*F*(1, 24) = 7.2, *p* = .013, η_p_^2^ = .23			*F*(1, 25) = 7.67, *p* = .01, η_p_^2^ = .23
Design		*F*(1, 23) = 12.19, *p*<.002, η_p_^2^ = .35				
Old/New x Criterion		***F*** **(1, 23) = 8.8, ** ***p*** **<.007, η_p_^2^ = .28**				***F*** **(1, 25) = 9.29, ** ***p*** **<.006, η_p_^2^ = .27**
Old/New x Design	*F*(1, 23) = 5.15, *p*<.033, η_p_^2^ = .18					
Criterion x Design						
Old/New x Criterion x Design				***F*** **(2, 48) = 3.26, ** ***p*** **<.05, η_p_^2^ = .12 ** ***Post hoc*** **: Cons_LowFam > Cons_New**		

*Note*: Only significant results are shown. Greenhouse-Geisser corrected p-values are reported for effects in Experiment 2. Highlighted are late interactions of old/new with criterion. For electrode locations, see Methods section of Experiment 1.*Experiment 2 contained factor Condition with the three levels HighFam (Old items with high familiarity), LowFam (Old items with low familiarity), and New (new items). Effects were entered in the Old/New row when HighFam and LowFam were both significantly different from New. Effects were entered in the Old/New x TF row when HighFam was significantly different from LowFam in addition to LowFam being significantly different from New.

**Table 2:** Results of the ANOVA of **parietal** ERP amplitudes.

Experiment	Experiment 1		Experiment 2*		Experiment 3	
Design (Factor)	Random/Block		Condition (HighFam, LowFam, New)		Familiarity (High/Low)	
**Time Window (ms)**	**Early (300–500)**	**Late (500–700)**	**Early (300–500)**	**Late (500–700)**	**Early (300–500)**	**Late (500–700)**
Old/New	*F*(1, 23) = 6.42, *p*<.002, η_p_^2^ = .22	*F*(1, 23) = 12.19, *p*<.002, η_p_^2^ = .35	*F*(2, 48) = 15.97, *p*<.001, η_p_^2^ = .40 *Post hoc*: (HighFam = LowFam) > New		*F*(1, 25) = 18, *p*<.001, η_p_^2^ = .42	*F*(1, 25) = 25.54, *p*<.001, η_p_^2^ = .51
Criterion (Lib/Con)						
Design						
Old/New x Criterion						*F*(1, 25) = 7.49, *p* = .011, η_p_^2^ = .23
Old/New x Design		*F*(1, 23) = 5.64, *p*<.027, η_p_^2^ = .20		*F*(2, 48) = 21.66, *p*<.001, η_p_^2^ = .47 *Post hoc*: HighFam > LowFam > New		
Criterion x Design						
Old/New x Criterion x Design	*F*(1, 23) = 7.53, *p*<.002, η_p_^2^ = .25					

*Note*: Only significant results are shown. Greenhouse-Geisser corrected p-values are reported for effects in Experiment 2. For electrode locations, see Methods section of Experiment 1. *Experiment 2 contained factor Condition with the three levels HighFam (Old items with high familiarity), LowFam (Old items with low familiarity), and New (new items). Effects were entered in the Old/New row when HighFam and LowFam were both significantly different from New. Effects were entered in the Old/New x TF row when HighFam was significantly different from LowFam in addition to LowFam being significantly different from New.

**Figure 4:**
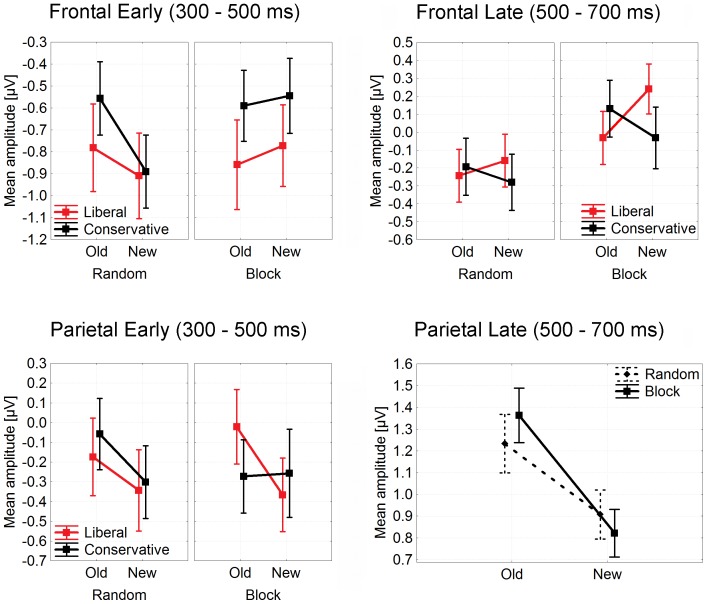
Mean ERP amplitudes taken in early (300–500 ms poststimulus) and late (500–700 ms poststimulus) time-windows at frontal (AFz, AF3, AF4, F1, F2, FCz, Fz) and parietal (see insert in Figure 3) sites in Experiment 1.

In the early time-window (300–500 ms poststimulus) at frontal ERP sites, there was a significant main effect of criterion as ERPs were less negative (more positive) going in the conservative condition relative to liberal. This effect reflected the same potential difference that has been described above for the frontocentral component FN320 (Figure 3), albeit with a slightly smaller effect size. There was also a significant interaction of old/new by block at these frontal sites, resulting from larger old/new differences in the random condition relative to block, where old/new differences were actually slightly reversed. At parietal sites, there were significant ERP old/new effects alongside a three-way interaction showing that the old/new effects were missing in the block condition for the liberal criterion. All these effects can be seen in the amplitude plots in Figure 4.

In the late time-window (500–700 ms poststimulus), ERPs showed a main old/new effect at parietal sites, plus an interaction of old/new with block, as old/new effects were larger in the blocked condition relative to random. At frontal sites (see Figure 4), old/new differences were almost absent in the blocked condition for the conservative criterion, and overall, amplitudes were less negative in the blocked condition, which led to a significant main effect of block and a significant old/new by criterion interaction.

### Discussion Experiment 1

The main goal of Experiment 1 was to assess ERP correlates of decision criteria in recognition memory judgments varied in line with experimental instructions either in an automatized, blockwise manner, or on a trial-by-trial randomized basis. We aimed to identify ERP correlates of, first, conservative as compared to liberal decision bias; and second, of automatized, habitual criterion-activation as compared to cue-dependent criterion shifts requiring the retrieval and maintenance of the cue as well as the flexible control of the decision bias on a given trial. Behavioral data showed that the latter condition was indeed more difficult relative to the automatized block condition as RTs were longer and the expected difference in the bias *Br* (liberal > conservative) was lower. The interesting question was how these variations would be reflected in the ERP correlates of memory and bias.

The ERP analysis revealed a frontocentral negative component (FN320) sensitive to the manipulation of decision criterion, with a smaller (i.e., more positive) amplitude for the conservative compared to the liberal condition. The direction and spatial distribution of this ERP difference was in line with our expectations, but the duration was relatively short. In absolute terms, the FN320 modulation was quite small (about 0.2 µV) but nonetheless reached a high significance level due to the high consistency across participants. Data of eight participants who did not comply with the criterion-setting instructions and who were excluded from the main analysis showed a tendency towards the *reversed* pattern (i.e., less negative FN320 for liberal compared to conservative (Figure S4). Although the component was generally not very clearly pronounced in that subsample, we think that this reversed amplitude difference further confirms our interpretation of the FN320 as a correlate (or part of a correlate) of bias.

Neither old/new effects nor the block/random manipulation modulated the FN320 in any significant way. However, these two factors, in parallel to criterion, did modulate frontal ERPs in the standard time-window of 300 to 500 ms poststimulus; with a pattern suggesting, first, higher positivity associated with a conservative criterion relative to liberal – a reflection of the same effect found in the FN320 – and secondly, positive old/new differences in the random condition compared to negative old/new differences in the block condition. At parietal sites, there were clearly significant old/new differences in all conditions except for the conservative bias in the block condition. Importantly, this difference between conservative and liberal was functionally different than that at the frontal sites as it reflected no main effect of criterion but a three-way interaction of criterion, block/random, and old/new item status.

In the late time-window of the standard analysis, frontal ERPs were less negative overall in the block condition relative to random, and effects of criterion interacted with ERP old/new differences as these were positive in the conservative condition and negative in the liberal condition. At parietal sites, ERPs showed stronger old/new effects in the block condition relative to random. We suggest that this mixture of interaction effects might reflect task-context effects; these appeared more complex at the frontal sites relative to parietal. Of particular interest is that retrieved old/new item information interacted with habitual (automatized) as compared to flexible (top-down controlled) criterion signals. At the parietal sites, the stronger old/new effects in the blocked condition compared to random suggest facilitated old/new differentiation based on controlled retrieval, in line with stronger effects in behavioral bias *Br* and shorter reaction times. However, late ERPs generally contain more variation within and between individuals due to variation in strategy and performance level compared with the more stimulus-driven early ERP correlates, so without any further support, we must maintain cautious about the interpretation of these effects purely in terms of task difficulty.

Taken together, the picture suggests that the FN320 component might be more specific to within-participant's variation of decision bias induced by instructions relative to the standard analysis, and more robust against the relatively strong experimental effects of automatizing versus flexible trial-by-trial control found in the behavior and the standard analysis. From the temporal pattern, it appears as if the FN320 might precede any task- or context-specific memory retrieval processes.

Notably, the effects of criterion found in the FN320 were main effects, with conservative less negative than liberal. This finding is in accord with our notion that the criterion reflects a threshold set on the memory strength dimension above which “old” responses are rendered and below which “new” responses are rendered. As the criterion is set at a higher level of familiarity in the conservative condition relative to liberal, its ERP correlate is a more positive potential, in line with the many ERP studies showing familiarity, memory strength, and retrieval confidence to increase ERP positivity. By contrast, the standard analysis that found interactions of old/new with other variables of task context, including criterion, is consistent with prior reports that reported interactions of bias effects with ERP old/new differences [35]–[37], suggesting an interaction of response bias processes with memory retrieval processes. To the degree that ERP old/new differences reflect accurate old/new discrimination, and not bias, such interactions are surprising, and inconsistent with the assumption of signal detection theory that discrimination performance and response bias are statistically independent, although this implication has not been discussed before in the existing ERP literature on bias.

We conducted Experiment 2 to more directly investigate the possibility that memory processes and criterion setting processes interact in our region of interest and in the standard ERP correlates of recognition memory.

## Experiment 2

In Experiment 2, we examined whether the bias manipulation by instruction would affect ERPs differently at two different levels of accurate old/new discrimination, to explore the possibility that criterion setting functions interact with memory retrieval processes. Decision criteria were manipulated by instruction in the same way as in the block condition of Experiment 1. In addition, we varied familiarity of the study items: Half of the old items were presented three times in the study phase to increase memory strength (high familiarity condition), half of them were presented only once as in any standard recognition memory task (low familiarity condition). ERPs between 300 to 500 ms are known to be sensitive to stimulus repetition effects, typically at posterior sites, while effects of perceived similarity occur at frontal sites [30]–[37], [46], [50], [57]. We thus expected the effects of the familiarity manipulation by stimulus repetition to be temporally overlapping with, but spatially separable from the effects of the bias manipulation.

### Materials and Methods Experiment 2

#### Participants

Data were obtained from 35 participants (students) who participated for course credit or a money reward of 20 Euro. All participants were healthy native German speakers with normal or corrected-to-normal vision and gave signed informed consent before participating in the experiment. Data of ten participants had to be excluded either due to an insufficient number of hits in one condition (conservative, low familiarity, n = 3) or due to large drift artefacts (n = 7). Thus, the final sample size consisted of 25 (20 females). The mean age was 24.9 years (range 19–43). All participants were right-handed.

#### Materials and procedures

Design and stimuli were the same as in Experiment 1 with the exception that the third factor (familiarity) was manipulated only in old items. Eight lists of words were created, each one containing a study list of 40 words of which 20 were shown once and 20 were repeated three times (in random order). Each test list contained 80 words (20+20 old and 40 new). Procedures, EEG-recordings and analyses were identical to Experiment 1. As a first step, we chose the same region and time window as in Experiment 1 for the ERP measures. In determining the behavioral indices *Br* and *Pr*, false alarm rates to new items were used for both, the high and the low familiarity conditions.

### Results Experiment 2

#### Behavioral data

**Figure 5:**
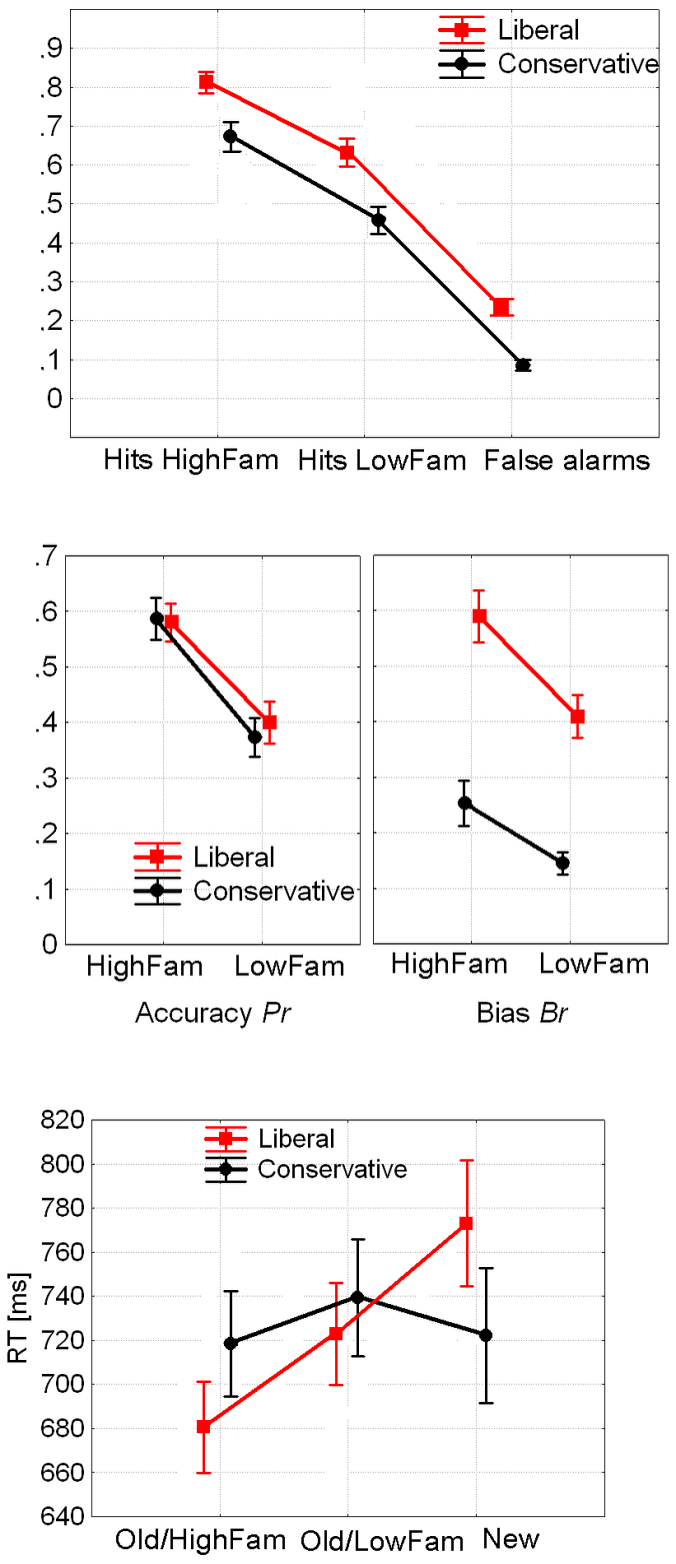
Hitrates (HR) and False Alarm Rates (FAR, top), Bias *Br* and Accurate Old/New Recognition *Pr* (center), and RTs (bottom) for Experiment 2 (error bars display standard errors). Note that *Br* and *Pr* are computed from the same False Alarm Rates for both levels of familiarity since familiarity was not manipulated in new items in this design (but see Experiment 3).

Figure 5 displays hit rates, false alarm rates, bias, accuracy, and RTs for Experiment 2. All 25 participants had higher HR and FAR in the liberal condition compared to the conservative condition. The two-way (Criterion x Familiarity) ANOVA of the bias measure *Br* revealed the expected main effect for Criterion (conservative < liberal; *F*(1, 24) = 94.2, *p*<.001, *eta^2^* = 0.8), in line with instructions, a main effect for Familiarity (low < high): *F*(1, 24) = 59.4, *p*<.001, *eta^2^* = 0.71, and a Criterion x Familiarity interaction: *F*(1, 24) = 6.25, *p*<.02, *eta^2^* = 0.21, indicating a larger familiarity effect in the liberal condition relative to conservative. The analysis of accurate old/new recognition *Pr* revealed a main effect of Familiarity (low < high): *F*(1, 24) = 239.2, *p*<.001, *eta^2^* = 0.91, as the high familiarity condition was easier, as expected, and a Criterion x Familiarity interaction: *F*(1, 24) = 4.74, *p*<.04, *eta^2^* = 0.16, indicating for the low familiarity condition a larger difference liberal vs. conservative.

As in Experiment 1, *Br* and *Pr* correlated highly (average *r* = .94) with SDT indices of bias and accuracy, respectively, as detailed in Table S1.

Mean RTs for correct responses were analyzed by an ANOVA of the two within-subjects factors Condition with three levels (old-low-familiarity, old-high-familiarity, new) and Criterion (liberal/conservative). A significant interaction showed RTs to be higher for new items in the liberal condition compared to all other conditions; *F*(2, 48) = 28.2, *p*<.001, *eta^2^* = 0.54.

#### ERP data

**Figure 6:**
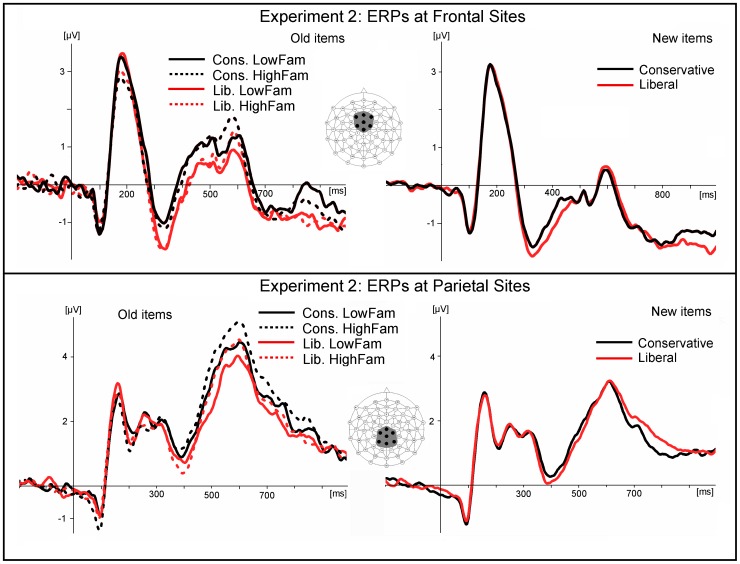
Frontocentral grand average ERPs for old (left) and new (right) items of Experiment 2. The negative FN320 component peaking around 320 ms was larger (more negative) for the liberal than for the conservative decision criterion. BOTTOM: Parietal grand average ERPs for the random condition (left) and the block condition (right) of Experiment 2. The displayed waveforms are averaged across the seven sites included in the statistical analysis (see inserts).

As in Experiment 1, a frontocentral FN320 peaking around 320 ms with a maximum at FCz was apparent (Figure 6). The ANOVA of the repeated measures factors Electrode Site, Criterion (liberal, conservative) and Condition (with the three levels old-low-familiarity, old-high-familiarity, new) performed on mean ERP amplitudes taken at 300–350 ms poststimulus at the seven frontocentral sites revealed main effects for Electrode Site: *F*(6, 144) = 2.72, *p*<.02, *eta^2^* = 0.10, and for Criterion: *F*(1, 24) = 5.95, *p*<.023, *eta^2^* = 0.20. As in Experiment 1, the component was larger (i.e., more negative) in the liberal compared to the conservative condition (for both, old and new items). A significant effect of Condition was found as well: *F*(2, 48) = 4.33, *p*<.022, *eta^2^* = 0.15. The post-hoc comparison revealed a significantly more positive FN320 amplitude for old items (both high and low in familiarity) compared to new. No significant interaction of Criterion x Condition was obtained (*p* = .44).

It should be noted that in contrast to Experiment 1, the ERP difference between the liberal and conservative conditions did not disappear after 350 ms but maintained until 460 ms poststimulus for new items, and extended even until about 600 ms poststimulus for old items (see Figures 6 and S2).

**Figure 7:**
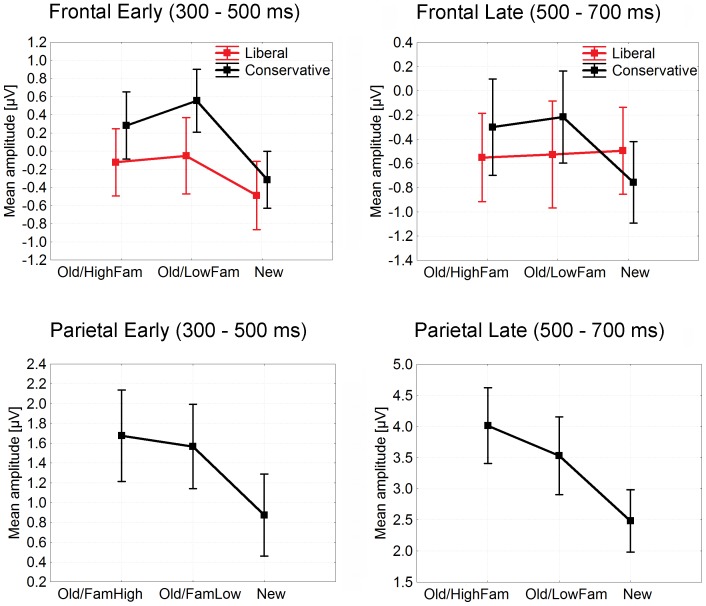
Mean ERP amplitudes taken in early (300–500 ms poststimulus) and late (500–700 ms poststimulus) time-windows at frontal (Fz, AFz, AF3, AF4, F1, F2, FCz) and parietal (see insert in Figure 6) sites in Experiment 2.

The standard analysis of ERP old/new effects (detailed in Tables 1 and 2) showed the same criterion effect that is reflected in the FN320, albeit temporally more extended, at frontal sites in the early time-window (300–500 ms poststimulus): The conservative criterion was associated with more positive going ERPs relative to the liberal, with a similar effects size as in the FN320 (*eta^2^* = .20). In addition, the factor condition was significant for which post hoc tests indicated that old items (both high and low in familiarity) were significantly more positive going than were new items (see amplitude plots in Figure 7). That same effect of condition was also significant for early parietal ERPs, albeit without any significant main effect of criterion (hence criterion is not differentiated in Figure 7).

In the late time-window (500–700 ms poststimulus), the pattern was slightly different for the liberal condition: Frontal ERPs showed the significant old/new effect only for the conservative criterion (interaction of Condition x Criterion). At parietal ERP sites, there was again a main effect of condition; post hoc tests indicated that old items of high familiarity were significantly more positive going than were old items of low familiarity which, in turn, were significantly more positive going than new items. Hence the analysis of late parietal ERPs is the only one where effects of induced memory strength (high versus low familiarity) became significant. No other effects or interaction effects were significant.

### Discussion Experiment 2

Behavioral data (hit rates and false alarm rates) showed that the manipulation of the decision criterion was again successful. Furthermore, higher hit rates and shorter RTs for high familiarity in comparison to low familiarity study items showed that the manipulation of memory strength was successful as well. This led to higher old/new recognition accuracy in the high familiarity condition, but also to a more liberal bias, consistent with earlier studies [39], [47].

Confirming the results of Experiment 1, the frontocentral FN320 component was modulated by the decision criterion; it was smaller (more positive) in the conservative condition relative to the liberal condition. Unlike in Experiment 1, however, this effect extended over time until about 450 ms poststimulus, and was distributed more anteriorly, where it became significant in frontal ERPs taken in the early time-window of 300–500 ms poststimulus, grossly in line with previous reports [37].

In these early frontal ERPs, we did not find any differential effect of bias at high versus low levels of familiarity, suggesting that these ERP correlates were independent of our manipulation of memory strength. As found in Experiment 1, the conservative criterion was generally associated with more positive amplitudes relative to the liberal criterion, an effect that was visible in both, ERPs to old items (hits) and ERPs to new items (correct rejections; see Figure 6). This independence of ERP bias effects from early ERP old/new differences conforms to the theoretical understanding of decision bias being statistically independent of memory for studied items.

However, effects of bias did interact with old/new differences in the late time-window (500–700 ms poststimulus), when old/new effects became larger for the conservative condition relative to liberal at frontal ERP sites, albeit being still insensitive to the effects of familiarity (i.e., memory strength). At parietal sites, ERPs were sensitive to the familiarity of the studied items, in line with earlier reports where list strength was manipulated [39]. This difference emerged in both bias conditions.

We conclude that early ERP effects of bias occurred at frontal sites consistently and independently of memory strength effects across the two investigated levels of item familiarity, and more sustained so than in Experiment 1, possibly because task conditions allowed participants to maintain an automatized criterion across blocks of trials in all conditions. However, as in Experiment 1, bias effects interacted with ERP old/new effects *late* during the recording epoch at frontal sites, where ERP old/new differences were more sensitive to study status in the conservative condition relative to liberal. We speculate that participants gave priority to the setting of the criterion during initial processing of the test stimuli, and focused on familiarity only later on during the trial, presumably by involving controlled retrieval processes.

## Experiment 3

To further test and challenge ERP correlates of bias, we used another manipulation that is suitable for manipulating the bias via bottom-up driven familiarity, meaning to affect early ERP indices of memory retrieval. While maintaining the participant-driven manipulation of bias by blockwise instruction, as in Experiments 1 and 2, participants' decision-making was additionally manipulated on a trial-by-trial basis, albeit this time not through subject-driven control processes as in Experiment 1, but through bottom-up driven familiarity effects, as in Experiment 2. However, contrary to Experiment 2, this memory manipulation was meant to affect only the bias, not accurate old/new discrimination, and to specifically target automatic, involuntary retrieval from memory.

To that end, we induced high familiarity in both, old and new items, thereby inducing memory intrusions. We preexposed participants in a separate reading task performed prior to the study phase of the recognition memory task to half of the test items, of which half were presented in the subsequent study phase of the recognition memory task (old test words), and half were not (new test words). In the subsequent recognition memory test, recognizing prestudied old test words as “old” is relatively easy as the preexposure automatically boosts feelings of familiarity. However, recognizing prestudied *new* words (that occurred in the preexposure phase but not in the study phase) as “new” is difficult as the preexposure would make these items feel old, leading to inadvertent memory intrusions unless the source of the oldness feeling is clearly distinguished. As a consequence, while the preexposure manipulation increases hit rates (“old” responses to old items), it also increases false alarm rates (“old” responses to new items). The manipulation thus increases the bias to respond “old” by increasing familiarity through preexposure – without affecting accurate old/new recognition performance.

Notably, contrary to the coincident manipulation of bias by task instruction, which can only be top-down controlled by participants (albeit automatized over blocks of trials), the bias manipulation by preexposure induces a *stimulus-driven* retrieval bias due to enhanced item fluency and accessibility [38], [48], [49]. In comparing the high familiarity (preexposure) and the low familiarity (no preexposure) conditions in the conservative and liberal conditions, respectively, this task design allowed us to investigate the joint influence of two qualitatively different types of manipulations of bias on ERPs, both of which are based on automatic processes. We reasoned that the two manipulations might result in additive effects on early frontal ERP correlates, with the largest (most negative) potential in the liberal-high familiarity condition, and the smallest (least negative) potential in the conservative-low familiarity condition.

### Materials and Methods Experiment 3

#### Participants

Data were obtained from 35 participants (students) who participated for course credit or a monetary reward of 20 Euro. All participants were healthy native German speakers with normal or corrected-to-normal vision and gave signed informed consent before participating in the experiment. Data of eight participants had to be excluded due to an insufficient number of hits in at least one condition (conservative, low familiarity) for ERP analysis which led to noisy data. Another participant was excluded due to excessive eye blink artefacts which could not be corrected. Thus, the final sample size was 26 (19 females). The mean age was 22.1 years (range 19–34), and 24 participants were right-handed.

#### Stimuli and stimulation sequences

Word stimuli and list construction were the same as in Experiments 1 and 2. To induce familiarity by preexposure, half of the old and half of the new test items were presented twice in a prior reading task performed before the recognition memory task. Four lists for the liberal condition and four lists for the conservative condition were created. Each list contained a study block of 40 words and a recognition block of 80 words. These 80 words were assigned equally to the following conditions (i) old-high familiarity (studied items with preexposure). (ii) old-low familiarity (studied items without preexposure). (iii) new-high familiarity (unstudied items with preexposure), (iv) new-low familiarity (unstudied items without preexposure).

#### Experimental procedure

Participants were fully informed before the experiment about the task design, and were warned that words from the reading task (inducing familiarity via preexposure) would later serve as test probes in the recognition memory task. In the reading task itself, 160 words were presented twice in randomized order for 400 ms and an ISI of 1400 ms; half of these were later “old” items in the recognition test and the others “new”. Participants were instructed to press the left mouse button when they detected that an item was presented for the second time to ensure continuous attention. After the reading task, the study phase was run, followed by the recognition test phase, analogue to Experiments 1 and 2. For the recognition test, participants were explicitly instructed to ignore whether or not they had seen a test item in the reading task. Specifically, they were asked to render an “old” response only when a word had been presented in the study phase, the same procedures as in Experiments 1 and 2. The complete sequence (preexposure - study – memory test) was then repeated for the other response criterion. The instructions to respond liberal or conservative were identical to those of the previous experiments. The experimental procedures for the study and test phase, the EEG-recording and analyses as well as analysis of the behavioral data was identical to the block condition of Experiment 1.

### Results Experiment 3

#### Behavioral data

Figure 7 displays hit rates (HR), false alarm rates (FA), response bias *B_r_*, accurate recognition memory *P_r_*, and RTs in the four experimental conditions. The two-way ANOVA of the bias measure *Br* revealed the expected main effects for Criterion: *F*(1, 25) = 69.1, *p*<.001, *eta^2^* = 0.73, and for Familiarity: *F*(1, 25) = 64.7, *p*<.001, *eta^2^* = 0.72, plus a Familiarity x Criterion interaction: *F*(1, 25) = 9.83, *p*<.005, *eta^2^* = 0.28; due to larger effects of familiarity in the liberal relative to the conservative condition. Only one participant in one condition (low familiarity) did not shift his/her bias in accord with the instructions; this participant was maintained as it had practically no effect on results.

Analysis of accurate old/new recognition memory *Pr* revealed only a significant interaction of Familiarity x Criterion: *F*(1, 25) = 11.6, *p*<.003, *eta^2^* = 0.32, showing reduced accuracy for high familiarity items in the liberal condition relative to the other conditions, suggesting that the high familiarity condition interfered somewhat with accurate old/new recognition when the decision criterion was liberal. In fact, the reason for both, the high bias and the low accuracy in this condition was the higher false alarm rate (see Figure 7).

As in the two other experiments, *Br* and *Pr* correlated highly (average *r* = .92) with SDT indices of bias and accuracy, respectively (details in Table S1).

Mean RTs for correct responses were analyzed using an ANOVA of the three within-subjects factors Response Type (old/new), Criterion (liberal/conservative), and Familiarity (low/high). A main effect of Familiarity revealed, on average, faster (18 ms) responses for low- vs. high familiarity items: *F*(1, 25) = 21.1, *p*<.001, *eta^2^* = 0.46. Two interactions were significant: Response type x Criterion: *F*(1, 25) = 25.3, *p*<.001, *eta^2^* = 0.5, and Response type x Familiarity: *F*(1, 25) = 86.3, *p*<.001, *eta^2^* = 0.78. As Figure 7 shows, the main reason for the faster responses in the low familiarity condition was the high RTs to new items in the high familiarity condition where the expected memory intrusions occurred. Conversely, new items in the low familiarity conditions were distinctly new compared to all other conditions and were therefore easily identified, which is why responses to these items were fastest.

#### ERP data

**Figure 8:**
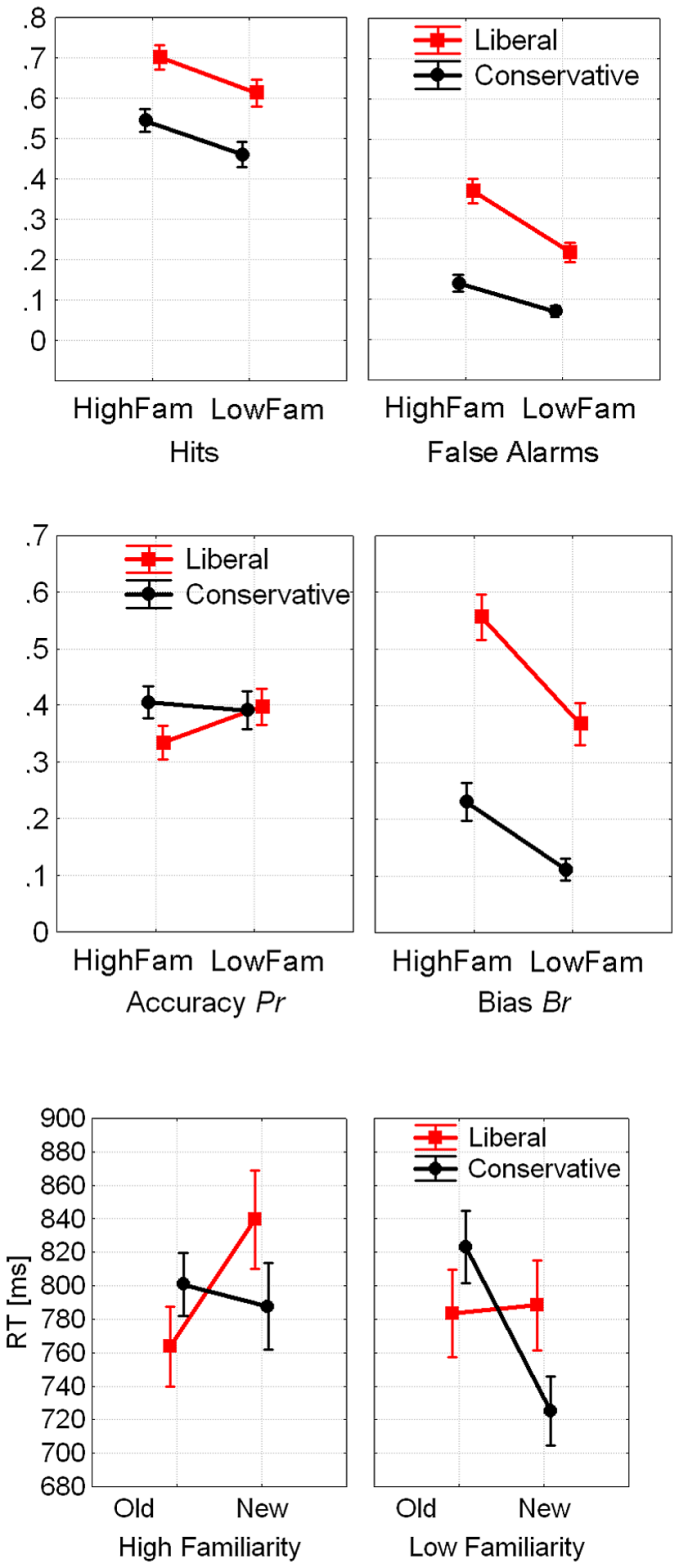
Hitrates (HR) and False Alarm Rates (FAR, top), Bias *Br* and Accurate Old/New Recognition *Pr* (center), and RTs (bottom) for Experiment 3 (error bars display standard errors).

As in Experiments 1 and 2, the grand average waveform showed a frontocentral negativity peaking around 320 ms with a maximum at FCz (FN320). In the low familiarity condition that reflects a standard old/new recognition memory task, this component was smaller (more positive) in the conservative compared to the liberal condition, and larger for new than for old items. In the high familiarity condition with the bottom-up increased bias, no such differentiation could be observed (Figure 8).

**Figure 9:**
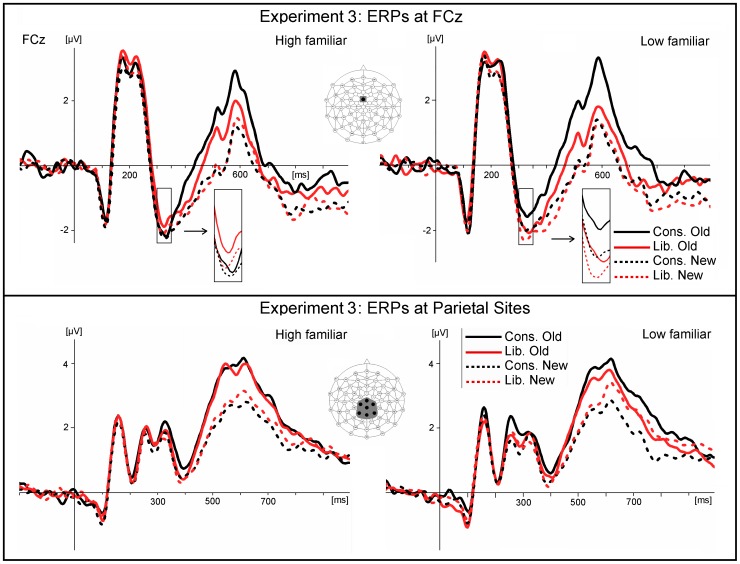
Frontocentral (site FCz) grand average ERPs for high familiarity items (left) and the low familiarity items (right) of Experiment 3. The FN320 was larger (more negative) for the liberal than for the conservative decision criterion only in the low familiarity condition. In the later time window around 600 ms poststimulus, the ERP old/new differences were larger in the conservative condition than in the liberal condition. BOTTOM: Parietal grand average ERPs for the low familiarity (left) and the high familiarity condition (right) of **Experiment 3**. The parietal waveform was averaged across the seven sites included in the statistical analysis (see insert).

An ANOVA (mean amplitude 300–350 ms poststimulus) was performed with the within-subjects factors Old/New, Criterion (liberal, conservative), Familiarity (High, Low), and the seven electrode sites (FCz and the surrounding sites Fz, Cz, F1, F2, FC1, FC2). Apart from a significant old/new effect, *F*(1, 25) = 4,46, *p*<.05, *eta^2^* = 0.15, the analysis showed that the interaction of Criterion x Familiarity was marginally significant, *F*(1, 25) = 3.31, *p* = .08, *eta^2^* = 0.12, and so was the four-way interaction with electrode site: *F*(6, 150) = 2.07, *p*<.10. *eta^2^* = 0.08. More detailed inspection suggested that main effects of bias were evident only at electrode sites Fz and FCz in the low familiarity condition. An ANOVA restricted to FCz, where the effect of bias was largest, indeed revealed a significant interaction of Criterion x Familiarity: *F*(1, 25) = 8.33, *p*<.01. *eta^2^* = 0.25, in addition to a significant effect of Old/New: *F*(1, 25) = 4.98, *p*<.035, *eta^2^* = 0.17. The post hoc test revealed a significant difference in the FN320 amplitude (liberal more negative than conservative) in the low familiarity condition that was absent in the high familiarity condition (Figure 9). Thus, the marginal effects described for the entire ROI turned out significant when only the center of the ROI was considered, and was due to a significant effect of bias on the FN320 in the low familiarity condition only. The grand average depicted in Figure 9 shows that this ERP difference (conservative more positive than liberal for both, old and new items) maintained apparent until about 450 ms poststimulus.

**Figure 10:**
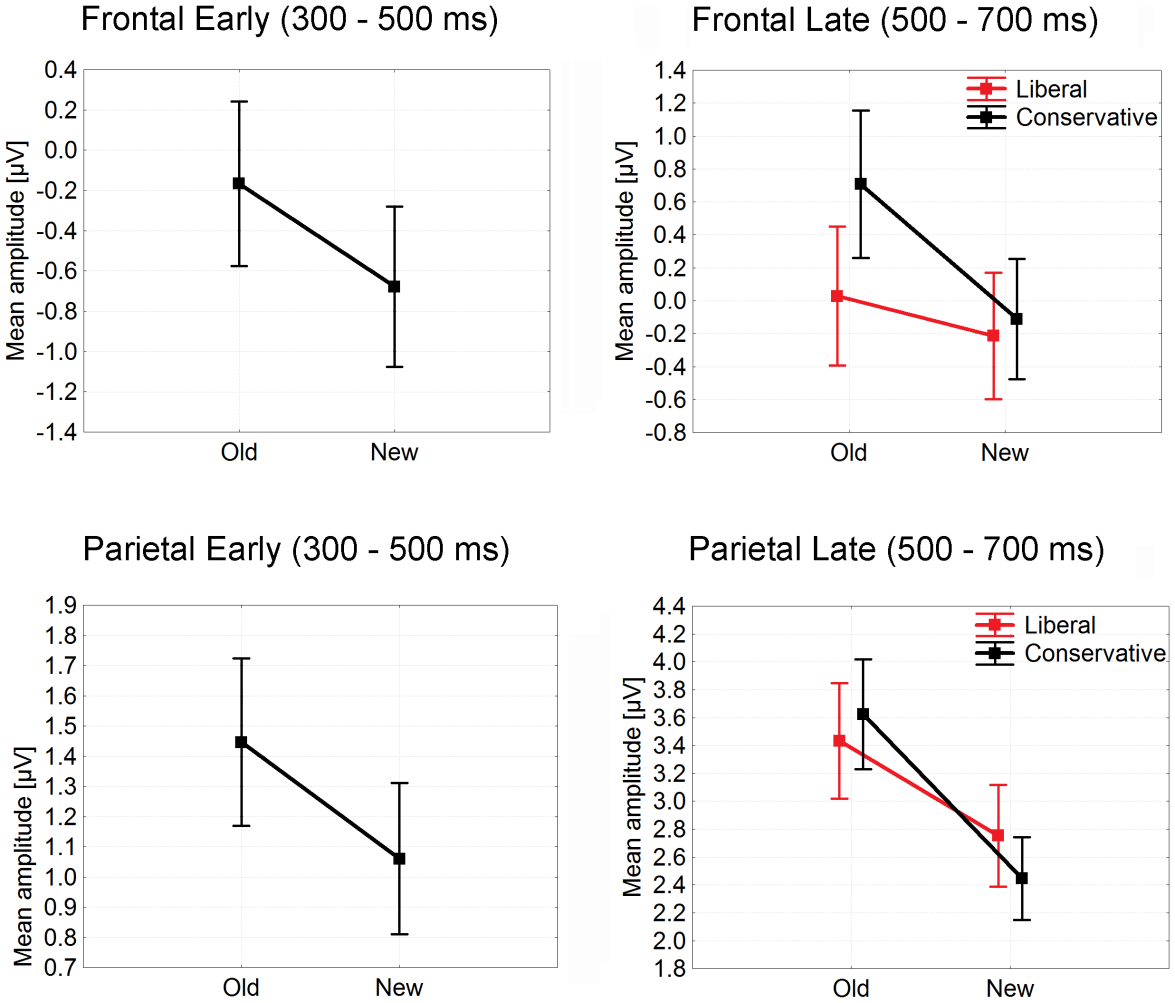
Mean ERP amplitudes taken in early (300–500 ms poststimulus) and late (500–700 ms poststimulus) time-windows at frontal (Fz, AFz, AF3, AF4, F1, F2, FCz) and parietal (see insert in Figure 9) sites in Experiment 3.

In the standard ERP analyses (detailed in Tables 1 and 2), the early time-window (300–500 ms poststimulus) revealed only significant old/new effects at both, frontal and parietal sites. However, ERPs from the late time-window (500–700 ms poststimulus) showed, first, a significant main effect of bias at frontal sites as potentials were more positive in the conservative condition relative to liberal, and second, significant interactions of criterion with old/new effects at both frontal and parietal sites; in both these cases, old/new differences were larger for the conservative condition relative to liberal (see Figure 10).

### Discussion Experiment 3

Familiarity of the recognition test materials was manipulated via preexposure to induce a memory retrieval bias that would interact with early effects of instructed decision bias expected to manifest in frontal ERPs. In the behavioral responses, we found, as expected, shifts of the decision criterion as a function of both, instruction and familiarity. Items that had been presented before the recognition memory task were more “fluent” due to the repetition and therefore retrieved more automatically [38], [48]–[50]. As expected, this led to more “old” responses in response to both, old and new test items, thereby increasing the response bias (i.e., rendering the criterion for “old” responses more liberal). In addition, behavioral data showed that participants set and maintained their response criterion in accordance with the bias instructions. Hence their response criterion on every single trial was jointly determined by a tonic (blockwise by instruction) and a phasic (trialwise by familiarity) component. By contrast, and in contrast to Experiment 2, accurate old/new recognition was largely unaffected by these manipulations.

As in Experiments 1 and 2, early ERP indices of instructed bias effects were found in the FN320, but this time only in the low familiarity condition which reflects the standard old/new recognition memory test condition. However, even here, effects were weaker than in the other two experiments as they became only marginally significant for the entire frontocentral cluster, and significant only at the central frontal site (FCz). In the high familiarity condition, effects involving bias were far from significant (*p*>.90). This high familiarity condition is the one where subject-driven and stimulus-driven bias effects were expected to mix (or add up), and participants had to carefully monitor the true source of any “oldness” feelings while being tempted to mistakenly classify new items as “old” [37]. Perhaps these circumstances have diminished the ERP correlates of bias in the FN320, and at the same time slowed down responses. This interpretation is consistent with traces of familiarity effects visible at frontal and frontocentral sites in the T-maps between 200 and 500 ms poststimulus (Figure S2). However, since none of the planned statistical analyses proved any early effects of familiarity to be significant, these interpretations are speculative.

The standard analyses of ERPs in the late time-window showed a significant main effect of bias at frontal sites; again more positivity was associated with the conservative criterion. However, contrary to the finding in the FN320, this pattern was driven by enhanced positivity associated with old items only, which led to larger old/new differences in the conservative as compared to the liberal condition, so the main effect was secondary to the interaction. Larger old/new differences in the conservative condition compared to liberal were also found at parietal sites in this late time-window (see Figure 10). These findings are consistent with Experiments 1 and 2.

## General Discussion

The present study aimed at characterizing and specifying the effects of criterion-setting functions on ERPs recorded during recognition memory tasks. Based on prior studies [34]–[37], we hypothesized early frontal ERPs to be sensitive to bias effects. Our goal was to examine how sensitive, specific, and consistent across various task contexts the bias would be reflected in these correlates, and how criterion setting would interact with early and late accurate memory retrieval as indexed by standard ERP indices of recognition memory processes.

We performed three recognition memory experiments in which response criteria were manipulated within participants via instructions. Additionally, one other factor was varied in each experiment that was meant to challenge criterion-setting functions. In Experiment 1, this was the flexible trialwise shift of criterion-setting, in Experiment 2, the increased memory strength of studied items, and in Experiment 3, the induction of automatic retrieval bias by familiarity. In all three experiments, behavioral data indicated that our manipulations led to the expected effects, including longer reaction times for the trialwise criterion-shifts in Experiment 1, shorter reaction times for stronger memory traces in Experiment 2, and longer reaction times for source conflict resolution in Experiment 3.

ERP analyses of the three experiments yielded two central findings: the correlates of bias itself (main effects of bias) and the interaction of bias effects with old/new effects.

### Main effects of bias on ERPs

We found the FN320 to reflect instructed bias effects across all three experiments (with the exception of the high familiarity condition of Experiment 3). The component was consistently more positive for the conservative response criterion relative to liberal, i.e., when the trials involved in the ERP average contained relatively more sure “old” judgments. This ERP difference between conservative and liberal conditions had a frontocentral focus, and was temporally circumscribed to 300–350 ms in Experiments 1 and 3 (where in fact it occurred only in the low familiarity condition). In Experiment 2, where memory strength was varied in addition to decision criterion, and old/new discrimination was relatively easy (as shown by reaction times and accuracy), the difference lasted longer, until about 500 ms poststimulus for old items and about 450 ms for new items. Owing to this temporal variation, and to the markedly reduced effects seen in Experiment 3, we suggest that the FN320 is no “universal” marker of decision bias, but might mark the *onset* of criterion-setting processes, when these precede other task-related processes and are therefore relatively independent of task context.

Main effects of bias were less clear in Experiment 3, where significant effects of bias were spatially very focused, and emerged only at the peak of the FN320 in the low familiarity condition. As high and low familiarity items were randomly mixed, the recognition memory task in this experiment may have been more challenging than in the other two experiments. Specifically, automatic effects of induced familiarity, i.e., effects of retrieval bias, may have distorted and delayed the setting and maintenance of the decision criterion in this task context. In fact, standard ERP analyses failed to find any statistically reliable effects of bias in the early time-window.

Consistent with [37], any main effects of bias observed in the three experiments were consistently characterized by higher ERP positivity in the conservative condition compared to liberal at the frontal sites investigated, as should be expected if the decision threshold relates to a higher point of familiarity on the memory strength dimension (c.f., Figure 1). However, this result is inconsistent with [36] who reported the opposite pattern (higher positivity associated with liberal bias) for frontopolar sites, alongside larger early old/new effects for the conservative condition relative to liberal. The inconsistency is probably not only due to the different referencing (although different referencing alone can convert ERP differences at frontopolar and other inferior sites; c.f., note 4 on page 485 in [33]), but also to differences in the design of the comparisons. Specifically, in the study by [36], comparisons were made between individuals whereas in the present study (and in [37]), the bias was varied within individuals. That is, the criterion was determined by explicit instructions, and was not allowed to be set freely in accord with individual preferences. This might change the ERP correlates, because participants naturally tending towards a liberal bias are pressed to respond conservatively in the conservative condition, and participants naturally preferring a conservative bias are pressed to respond liberally in the liberal condition; in fact, all participants are prompted to shift their criteria in both bias conditions, thereby overruling their natural response tendencies. Notably, the frontopolar ERP correlates of free-floating bias observed in the prior study [36] varied immensely between high and low bias participants, and not just around 300–500 ms poststimulus, but across the entire recording epoch, even in N1, and likewise post decision, so that these possibly reflect general differences between individuals, and may be entirely unrelated to stimulus processing. Nonetheless, ERP correlates of bias may differ for between- and within-participant comparisons. At the moment, we solely refer to within-participant shifts of bias in suggesting that the FN320 might index initial criterion setting processes when the criterion is deliberately adapted to explicit task conditions (as opposed to stimulus-controlled or trait-controlled).

### Interaction effects of bias with ERP old/new effects

The second main finding of our study refers to interactions of ERP correlates of bias with ERP old/new effects. Whereas the above discussed bias effects on the FN320 and on frontal ERPs during the early time-window reflect main effects (“ERP conservative/liberal effects”, so to speak), without any significant impact on ERP old/new differences, we also found consistent evidence of interactions of bias with ERP old/new effects, across all three experiments. The main characteristic was that old/new differences were larger in the conservative relative to the liberal bias conditions, in line with earlier reports [34]–[37]. However, these interactions occurred later than the main effects of bias, so that they became significant only in the late time-window of the standard analyses. Depending on experiment, these interaction effects were significant at frontal (Experiments 1, 2, and 3) and parietal sites (Experiment 3), with parietal sites additionally showing other task effects (Experiments 1 and 2).

The temporal characteristic of the interactions of criterion with old/new item status suggests that the effects are related to controlled processes, not to “quick and dirty” automatic memory effects like familiarity. In addition to controlled memory retrieval [39], response monitoring processes could be involved that are typically associated with variations in late frontal ERPs [52]. In any case, the finding of larger old/new ERP differences for conservative compared to liberal criteria is not in line with SDT and 2 HTM ([1]–[3], [26], which both assume independence of bias and accuracy. Despite the parallels of this finding in prior studies [35]–[37], this theoretical implication has not been discussed before.

If old/new discrimination and bias were independent processes, then their brain correlates should not show any significant interactions. Perhaps the finding points to the limits of single process memory models as compared to dual process models: Whereas the former assume a single familiarity dimension underlying all memory signals, the latter propose a separate processing mode for episodic recollection, one that is quantitatively different in terms of latency and decision confidence, and qualitatively different in terms of neuronal basis and subjective experience (for a review, see [53]). Our ERP findings suggest that, when participants make old/new distinctions at relatively high levels of familiarity, as is the case in the conservative condition more so than in the liberal, they show more late recollection of studied item information (Experiment 3, frontal and parietal late ERPs), more sensitivity to the memory strength of test items (Experiment 2, frontal late ERPs), and higher capability to maintain positive old/new differences (Experiment 1, late frontal). This shows clearly that the conservative and the liberal thresholds are not equivalently operating regimes, but have different influences on memory retrieval processes, presumably because more recollection is involved in the conservative condition relative to liberal. In addition, the incidental finding of a longer duration of bias effects for old items relative to new items in Experiment 2 is not consistent with 2 HTM which assumes that old and new item recognition thresholds are equivalent. These theoretical implications need further investigation, preferably with ERPs because two-choice behavioral indices might be unable to show the distinctions outlined here.

### Study limitations

The designs of the experiments reported in this article are rather complex, investigating the effects of three experimental factors (Criterion, Old/New, one additional task design factor), plus Experiment (1 through 3) as an additional factor, on various electrode sites and time-windows. The complexity was necessary to compare effects of instructed bias in the three different memory task contexts on the relevant ERP correlates. However, future studies could limit their designs to classical manipulations of bias, e.g., by instructions, pay-off matrices, or by investigating individual differences, to focus on main effects of bias, before applying these indices to other contexts and interactions. Of particular interest would be the question of whether the FN320 is indeed specific to subject-driven variations of bias as opposed to bottom-up, input driven variations, as speculated here.

Another potential limitation of our study is that we could not provide a direct link between variations in behavioral indices of bias and the FN320. At first blush, one would expect any brain correlate of bias to be correlated with the behavioral index of that same cognitive function. In the present study, we did find a significant covariation of FN320 with *Br* in Experiment 1 when all participants were reconsidered (including the eight participants that were excluded from the main analysis as they had not complied with the bias instructions), but not in the other two experiments where no such cases occurred. Hence, limited variance may be one explanation for the lacking covariation. Remarkably, however, correlations between ERP measures and behavioral performance are almost never reported in the recognition memory literature, in sharp contrast to the functional magnetic imaging literature where such analyses are standard. One reason may lie in the very high individual variability of ERP average waveforms; ERP components are known to vary considerably in size between individuals. Experimental effects might covary with the individual size of the ERP components, such that large amplitudes may be prone to larger experimental effects, in which case the ERP indices could be entirely uncorrelated with behavior. The issue is certainly worth systematic and extensive investigation, but beyond the scope of this report.

### Summary and conclusions

What does our results pattern mean for the dynamics of recognition memory judgments? We suggest that, during any ordinary recognition memory trial (one that only asks for quick identification of old items), initially a threshold is activated for “old” responses at or around 300 ms poststimulus, that is reflected in the FN320, and that is then compared with retrieved memory signals. If the retrieved memory information exceeds the threshold, an “old” response is rendered; if it falls short of the threshold, a “new” judgment is rendered. The equivalent happens when new items are defined as the target. For items with a relatively large distance to the decision threshold, the decision can be made easily; for other items whose familiarity lies relatively close at the decision threshold, the decision requires more controlled analysis. We suggest that it is these latter processes that drive interactions of ERP conservative/liberal differences with ERP old/new differences as these occur late during the recording epoch.

The idea of criterion setting as one of the first processes taking place, even before any controlled memory retrieval sets in, may be surprising to researchers who implicitly or explicitly understand criterion setting as a metacognitive process that is performed if and only if (i.e., only *after*) accurate memory retrieval fails [51], in line with the decision tree depicted for 2 HTM in Figure 1. However, the proposal fits well, first, with prior data [34]–[37], and second, with the general idea that the brain routinely enacts preparatory sets to be able to respond quickly in case of uncertainty, ambiguity, and conflict [54], [55], as proposed for instance by the memory prediction framework [56]. According to our data, the criterion activation occurs as early as 300 ms poststimulus, quite independent of memory task requirements (with the exception of a criterion that is additionally controlled by stimulus-driven retrieval bias; as in the high familiarity condition of Experiment 3). This is before or coincident with the onset of implicit memory retrieval [32],[57], and is integrated after 500 ms poststimulus with retrieved memory information and other task requirements. The latter processes may involve executive control like task switching [58]–[60], as in Experiment 1, or source retrieval [61], as in Experiment 2.

Does the setting of the threshold itself require top-down executive control? We think that it does to the degree that the setting of the threshold relates to a hitherto unpracticed process in the service of goal-attainment. However, once the setting of the threshold becomes automatized, as in the block conditions of our experiments, executive control is no longer needed. Likewise, in comparisons between individuals freely setting their response thresholds (i.e., comparisons of individuals who habitually show reduced cognitive control (e.g., individuals high in impulsivity) with those who habitually show high cognitive control), for as long as both groups of individuals are not required to adjust their decision thresholds flexibly in line with current task demands, the threshold-setting process should be an automatic process that does not require any extra intervention by executive control. In fact, in this case the process might be better referred to as “criterion retrieval” or “criterion activation” as it does not involve any selection, definition, or evaluation of a criterion, but only its activation on a given trial. According to our understanding, this still reflects a gating function, a function with an early onset that determines the ease by which retrieved item information is transformed into a particular behavioral response, and that is subject to executive control only when strategic adjustments are required to pursue current goals.

Further research must clarify how “universal” early effects of bias on frontal ERPs are, especially under conditions of added retrieval bias (Experiment 3). Quite possibly, the correlate might occur later or temporally less circumscribed when criterion setting is influenced (“biased”) by stimulus-driven information. In fact, [51] found response bias (manipulated via payoff matrices) to be reflected not in early, but in late parietal and even postresponse ERPs, and interpreted these as metacognitive processes rather than as an early “gating function” as we do. In that study, the to-be-adopted bias was not known a priori by participants but had to be extracted from payoff-matrices, and therefore was not top-down controlled, or at least not initially. Likewise, we think that highly speeded tasks might yield an ERP correlate of bias that occurs even earlier than 300 ms poststimulus (e.g., 11, [62]).

In sum, we conclude that ERP conservative/liberal differences can and should be used to track response criterion setting in standard recognition memory tasks. At the least, they can be used to determine bias as a potential confound in ERP investigations of memory processes, particularly when source monitoring or familiarity is involved, as these processes affect potentials with similar temporal and spatial distributions [63], [64]. In addition, ERP indices of bias might stimulate research that helps clarifying ongoing discussions about proper modeling of memory decisions [16]–[25]. Contrary to behavioral indices, ERPs are free of statistical or theoretical assumptions and sensitive to the timing of the underlying cognitive processes, and can therefore help to decide between alternative accounts of memory phenomena such as false memories [15], the revelation effect [65], or list strength effects [39].

## Supporting Information

Figure S1 **Statistical difference maps (t-values) of ERPs recorded in Experiment 1 for comparisons of old versus new items (Old/New), liberal versus conservative criterion (Lib/Con), random versus block conditions (Rnd/Block), and the two- and three way interactions of these differences.** Interaction effects were determined as differences between differences, and then tested against zero. Mean t-values of the sample-by-sample t-test were calculated for the time-windows specified at the bottom. For a better illustration of the significant effects the scaling was set to −2/+2 (corresponding to a t-value of approximately *p* = .05, uncorrected for multiple testing).
(TIF)

Figure S2 **Statistical difference maps (t-values) of ERPs recorded in Experiment 2 for comparisons of old versus new items (Old/New), liberal versus conservative criterion (Lib/Con), highly familiar old items versus lowly familiar old items (Familiarity Old Items), and the two-way interactions of these differences.** Mean t-values of the sample-by-sample t-test were calculated for the time-windows specified at the bottom. For a better illustration of the significant effects, the scaling was set to −2/+2 (corresponding to a t-value of approximately *p* = .05, uncorrected for multiple testing).
(TIF)

Figure S3 **Statistical difference maps (t-values) of ERPs recorded in Experiment 3 for comparisons of old versus new items (Old/New), liberal versus conservative criterion (Lib/Con), highly familiar items versus lowly familiar items (Familiarity), and the two- and three way interactions of these differences.** Mean t-values of the sample-by-sample t-Test were calculated for the time-windows specified at the bottom. For a better illustration of the significant effects the scaling was set to −2/+2 (corresponding to a t-value of approximately *p* = .05, uncorrected for multiple testing).
(TIF)

Figure S4 **Mean ERP amplitudes (time-window 300–350 ms poststimulus) of the frontocentral negativity (FN320) in Experiment 1 shown separately for** ***N*** **= 24 participants who varied their decision criterion in accordance with instructions (good performers) and** ***N*** **= 8 subjects who did not comply with the instructions (poor performers) in at least one of the experimental conditions.** An ANOVA with the between-subjects factor Group and the repeated measures factors Electrode Site, Block (block/random), Criterion (liberal/conservative), and Old/New revealed the following significant effects: First, an interaction of Old/New x Block: *F*(1, 30) = 9.15, *p* = 0.0051, *eta^2^* = 0.23 yielding old > new differences in the random condition that were reversed in the block condition, and secondly, an interaction of Group x Criterion: *F*(1, 30) = 20.68, *p*<0.0001, *eta^2^* = 0.41, indicating a larger FN320 bias effect (liberal more negative than conservative) for the good performers that was reversed in the group of poor performers.
(TIF)

Table S1 **Pearson's correlations of behavioral indices of Two-High-Threshold Model with indices of parametric and nonparametric Signal Detection Theory (SDT).**
(DOCX)

## References

[1] Green DM, Swets JA (1966) Signal detection theory and psychophysics. Huntington: Robert E. Krieger Publishing Co.

[2] Macmillan NA, Creelman CD (2005) Detection theory: A user's guide (2nd ed.). Mahwah: Lawrence Erlbaum Associates.

[3] Erdfelder E, Brand M (2007) Memory measurement. In: Wassmann, J, Stockhaus K, editors. Experiencing New Worlds. New York: Berghahn Books. pp. 201–223.

[4] Zhang J, Mueller ST (2005) A note on ROC analysis and non-parametric estimate of sensitivity. Psychometrika 70: 203–212 10.1007/s11336-003-1119-8

[5] Stanislav H, Todorov S (1999) Calculation of signal detection theory measures. Behav Res Methods Instrum Comput 31: 137–149. 10495845 10.3758/bf03207704

[6] Verde MF, Macmillan NA, Rotello CM (2006) Measures of sensitivity based on a single hit rate and false-alarm rate: The accuracy, precision, and robustness of d′, Az, and A′. Percept Psychophys 68: 643–654. 16933428 10.3758/bf03208765

[7] Kantner J, Lindsay DS (2012) Response bias in recognition memory as a cognitive trait. Mem Cognit 40: 1163–1177 10.3758/s13421-012-0226-0 22872581

[8] De Jong P, Slaets PJP (2005) Response sets in self-report data and their associations with personality traits. Eur. J. Psychiat. 19: 209–214 10.4321/S0213-61632005000400002

[9] Wolf DH, Gerraty R, Satterthwaite TD, Loughead J, Campellone T, et al (2011) Striatal intrinsic reinforcement signals during recognition memory: relationship to response bias and dysregulation in schizophrenia. Front Behav Neurosci 5: 81 10.3389/fnbeh.2011.00081 22355285 PMC3280525

[10] Dougal S, Rotello CM (2007) “Remembering” emotional words is based on response bias, not recollection. Psychon Bull Rev 14: 423–429. 17874582 10.3758/bf03194083

[11] Reckless GE, Bolstad I, Nakstad PH, Andreassen OA, Jensen J (2013) Motivation alters response bias and neural activation patterns in a perceptual decision-making task. Neuroscience 238: 135–147 10.1016/j.neuroscience.2013.02.015 23428623

[12] Windmann S, Chmielewski A (2007) Emotion-induced modulation of recognition memory: Memory bias or response bias? Cogn Emot 22: 761–776 10.1080/02699930701507899

[13] Thapar A, Rouder JN (2009) Aging and recognition memory for emotional words: a bias account. Psychon Bull Rev 16: 699–704 10.3758/PBR.16.4.699 19648455

[14] Zhu B, Chen C, Loftus EF, Lin C, Dong Q (2013) The relationship between DRM and misinformation false memories. Mem Cognit 41: 832–838 10.3758/s13421-013-0300-2 23397226

[15] Dube C, Starns JJ, Rotello CM, Ratcliff R (2012) Beyond ROC curvature: Strength effects and response time data support continuous-evidence models of recognition memory. J Mem Lang 67: 389–406. 22988336 10.1016/j.jml.2012.06.002 PMC3442783

[16] Higham PA (2007) No special K! A signal detection framework for the strategic regulation of memory accuracy. J Exp Psychol Gen 136: 1–22. 17324082 10.1037/0096-3445.136.1.1

[17] Macmillan NA, Rotello CM, Verde MF (2005) On the importance of models in interpreting remember-know experiments: comments on Gardiner, et al.'s (2002) meta-analysis. Memory 13: 607–621. 16076675 10.1080/09658210444000269

[18] Miller MB, Wolford GL (1999) Theoretical commentary: The role of criterion shift in false memory. Psychol Rev 106: 398–405.

[19] Wixted JT, Stretch V (2000) The case against a criterion-shift account of false memory. Psychol Rev 107: 368–376. 10789201 10.1037/0033-295x.107.2.368

[20] Jang Y, Wixted JT, Huber DE (2009) Testing signal-detection models of yes/no and two-alternative forced-choice recognition memory. J Exp Psychol Gen 138: 291–306 10.1037/a0015525 19397385 PMC2789975

[21] De Carlo LT (2008) Process dissociation and mixture signal detection theory. J Exp Psychol Learn Mem Cognit 34: 1565–1572 10.1037/a0013081 18980416

[22] Pazzaglia AM, Dube C, Rotello CM (2013) A critical comparison of discrete-state and continuous models of recognition memory: Implications for recognition and beyond. Psychol Bull 139: 1173–1203. 23731174 10.1037/a0033044

[23] Batchelder WH, Alexander GE (2013) Discrete-state models: Comment on Pazzaglia, Dube, and Rotello (2013). Psychol Bull 139: 1204–1212 10.1037/a0033894 24188419

[24] Dube C, Rotello CM, Pazzaglia AM (2013) The statistical accuracy and theoretical status of discrete-state MPT models: Reply to Batchelder and Alexander (2013). Psychol Bull 139: 1213–1220. 24188420 10.1037/a0034453

[25] Malmberg KJ (2008) Recognition memory: a review of the critical findings and an integrated theory for relating them. Cogn Psychol 57: 335–384 10.1016/j.cogpsych.2008.02.004 18485339

[26] Snodgrass JG, Corwin J (1988) Pragmatics of measuring recognition memory: applications to dementia and amnesia. J Exp Psychol Gen 117: 34–50. 2966230 10.1037//0096-3445.117.1.34

[27] Kellen D, Klauer KC (2014) Discrete State and Continuous Models of Recognition Memory: Testing Core Properties under Minimal Assumptions. J Exp Psychol Learn Mem Cognit. In press. 10.1037/xlm0000016 24884647

[28] Bröder A, Kellen D, Schütz J, Rohrmeier C (2013) Validating a two-high-threshold measurement model for confidence rating data in recognition. Memory 21: 916–944 10.1080/09658211.2013.767348 23398213

[29] Rugg MD (1995) ERP studies of Memory. In: Rugg MD, Coles MGH, editors. Electrophysiology of the mind. Oxford: Oxford University Press. pp. 132–170.

[30] Allan K, Wilding EL, Rugg MD (1998) Electrophysiological evidence for dissociable processes contributing to recollection. Acta Psychol 98: 231–252. 10.1016/s0001-6918(97)00044-9 9621832

[31] Mecklinger A (2000) Interfacing mind and brain: A neurocognitive model of recognition memory. Psychophysiology 37: 565–582. 11037034

[32] Rugg MD, Curran T (2007) Event-related potentials and recognition memory. Trends Cogn Sci 11: 251–257. 17481940 10.1016/j.tics.2007.04.004

[33] Curran T, Tepe KL, Piatt C (2006) In: Zimmer HD, Mecklinger A, Lindenberger U, editors. Oxford: Oxford University Press.

[34] Windmann S, Kutas M (2001) Electrophysiological correlates of emotion-induced recognition bias. J Cogn Neurosci 13: 577–592. 11506658 10.1162/089892901750363172

[35] Windmann S, Sakhavat Z, Kutas M (2002b) Electrophysiological evidence reveals affective evaluation deficits early in stimulus processing in patients with panic disorder. J Abnorm Psychol 111: 357–369. 12003457

[36] Windmann S, Urbach TP, Kutas M (2002a) Cognitive and neural mechanisms of decision biases in recognition memory. Cereb Cortex 12: 808–817. 12122029 10.1093/cercor/12.8.808

[37] Azimian-Faridani N, Wilding EL (2006) The influence of criterion shifts on electrophysiological correlates of recognition memory. J Cogn Neurosci 18: 1075–1086. 16839282 10.1162/jocn.2006.18.7.1075

[38] Jacoby L (1991) A process dissociation framework: Separating automatic from intentional uses of memory. J Mem Lang 30: 513–514.

[39] Norman KA, Tepe K, Nyhus E, Curran T (2008) Event-related potential correlates of interference effects on recognition memory. Psychon Bull Rev 15: 36–43. 18605477 10.3758/pbr.15.1.36

[40] Oldfield R (1971) The assessment and analysis of handedness: The Edinburgh inventory. Neuropsychologia 9: 97–113. 5146491 10.1016/0028-3932(71)90067-4

[41] Hager W, Hasselhorn M (1994) Handbuch deutschsprachiger Wortnormen. Göttingen: Hogrefe.

[42] Lang PJ, Bradley MM, Cuthbert BN (2008) International affective picture system (IAPS): Affective ratings of pictures and instruction manual. Technical Report A-8. University of Florida, Gainesville, FL.

[43] Ferree TC, Luu P, Russell GS, Tucker DM (2001) Scalp electrode impedance, infection risk, and EEG data quality. Clin Neurophysiol 112: 536–544. 11222977 10.1016/s1388-2457(00)00533-2

[44] Picton TW, Bentin S, Berg P, Donchin E, Hillyard SA, et al (2000) Guidelines for using human event-related potentials to study cognition. Recording standards and publication criteria. Psychophysiology 37: 127–152. 10731765

[45] Bertrand O, Perrin F, Pernier JA (1985) A theoretical justification of the average reference in topographic evoked potential studies. Electroencephalogr Clin Neurophysiol 62: 462–464. 2415344 10.1016/0168-5597(85)90058-9

[46] Mecklinger A (2006) Electrophysiological measures of familiarity memory. Clin EEG Neurosci 37: 292–299. 17073167 10.1177/155005940603700406

[47] Verde MF, Rotello CM (2007) Memory strength and the decision process in recognition memory. Mem Cognit 35: 254–262. 10.3758/bf03193446 17645166

[48] Reggev N, Hassin RR, Maril A (2012) When two sources of fluency meet one cognitive mindset. Cognition 124: 256–260 10.1016/j.cognition.2012.04.001 22551704

[49] Windmann S, Krüger T (1998) Subconscious detection of threat as reflected by an enhanced response bias. Conscious Cogn 7: 603–633 10.1006/ccog.1998.0337 9817816

[50] Nessler D, Mecklinger A, Penney TB (2005) Perceptual fluency, semantic familiarity and recognition-related familiarity: an electrophysiological exploration. Brain Res Cogn Brain Res 22: 265–288. 15653299 10.1016/j.cogbrainres.2004.03.023

[51] Curran T, DeBuse C, Leynes AP (2007) Conflict and criterion setting in recognition memory. J Exp Psychol Learn Mem Cognit 33: 2–17 10.1037/0278-6393.33.1.2 17201551

[52] Johansson M, Mecklinger A (2003) The late posterior negativity in ERP studies of episodic memory: action monitoring and retrieval of attribute conjunctions. Biol Psychol 64: 91–117 10.1016/S0301-0511(03)00104-2 14602357

[53] Yonelinas AP (2001) Components of episodic memory: the contribution of recollection and familiarity. Philos Trans R Soc Lond B Biol Sci 356: 1363–1374. 11571028 10.1098/rstb.2001.0939 PMC1088520

[54] De Baene W, Brass M (2013) Switch probability context (in) sensitivity within the cognitive control network. Neuroimage 77: 207–214 10.1016/j.neuroimage.2013.03.057 23567890

[55] Hakun JG, Ravizza SM (2012) Cognitive control: preparation of task switching components. Brain Res 1451: 53–64 10.1016/j.brainres.2012.02.046 22444277

[56] Hawkins J (2004). On Intelligence. New York: Henry Holt.

[57] Rugg MD, Mark RE, Walla P, Schloerscheidt AM, Birch CS, et al (1998) Dissociation of the neural correlates of implicit and explicit memory. Nature 392: 595–598. 9560154 10.1038/33396

[58] Armbruster DJ, Ueltzhöffer K, Basten U, Fiebach CJ (2012) Prefrontal cortical mechanisms underlying individual differences in cognitive flexibility and stability. J Cogn Neurosci 24: 2385–2399 10.1162/jocna00286 22905818

[59] Hedden T, Gabrieli JD (2010) Shared and selective neural correlates of inhibition, facilitation, and shifting processes during executive control. Neuroimage 51: 421–431 10.1016/j.neuroimage.2010.01.089 20123030 PMC2852172

[60] Li L, Wang M, Zhao QJ, Fogelson N (2012) Neural mechanisms underlying the cost of task switching: an ERP study. PLoS One 7: e42233 10.1371/journal.pone.0042233 22860090 PMC3408496

[61] Turner MS, Simons JS, Gilbert SJ, Frith CD, Burgess PW (2008) Distinct roles for lateral and medial rostral prefrontal cortex in source monitoring of perceived and imagined events. Neuropsychologia 46: 1442–1453 10.1016/j.neuropsychologia.2007.12.029 18294660 PMC2697314

[62] Prime DJ, Jolicoeur P (2009) Response-selection conflict contributes to inhibition of return. J Cogn Neurosci 21: 991–999 10.1162/jocn.2009.21105 18752398

[63] Guillaume F, Tiberghien G (2013) Impact of intention on the ERP correlates of face recognition. Brain Cogn 81: 73–81. 23174431 10.1016/j.bandc.2012.10.007

[64] Mollison MV, Curran T (2012) Familiarity in source memory. Neuropsychologia 50: 2546–2565 10.1016/j.neuropsychologia.2012.06.027 22789677 PMC3432179

[65] Verde MF, Rotello CM (2004) ROC curves show that the revelation effect is not a single phenomenon. Psychon Bull Rev 11: 560–566. 15376811 10.3758/bf03196611

