# Supplementary figures and images for: Examining Event-Related Potential (ERP) Correlates of Decision Bias in Recognition Memory Judgments
Source: PLoS One. 2014 Sep 29;9(9):e106411. doi: 10.1371/journal.pone.0106411 (PMC4180069; doi:10.1371/journal.pone.0106411)

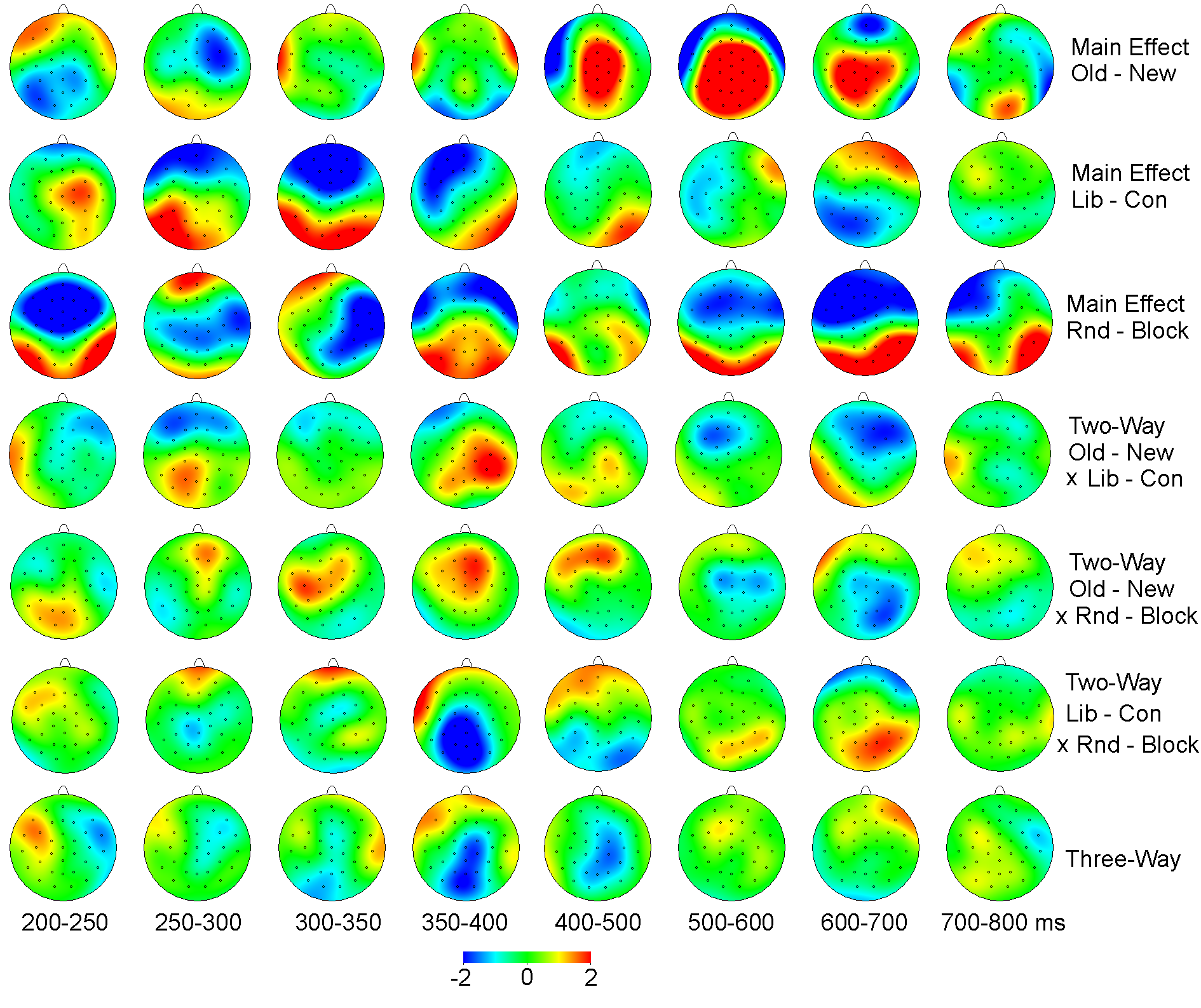

Supplement: Figure S1 — Statistical difference maps (t-values) of ERPs recorded in Experiment 1 for comparisons of old versus new items (Old/New), liberal versus conservative criterion (Lib/Con), random versus block conditions (Rnd/Block), and the two- and three way interactions of these differences. Interaction effects were determined as differences between differences, and then tested against zero. Mean t-values of the sample-by-sample t-test were calculated for the time-windows specified at the bottom. For a better illustration of the significant effects the scaling was set to −2/+2 (corresponding to a t-value of approximately p = .05, uncorrected for multiple testing). (TIF) [file pone.0106411.s001.tif]

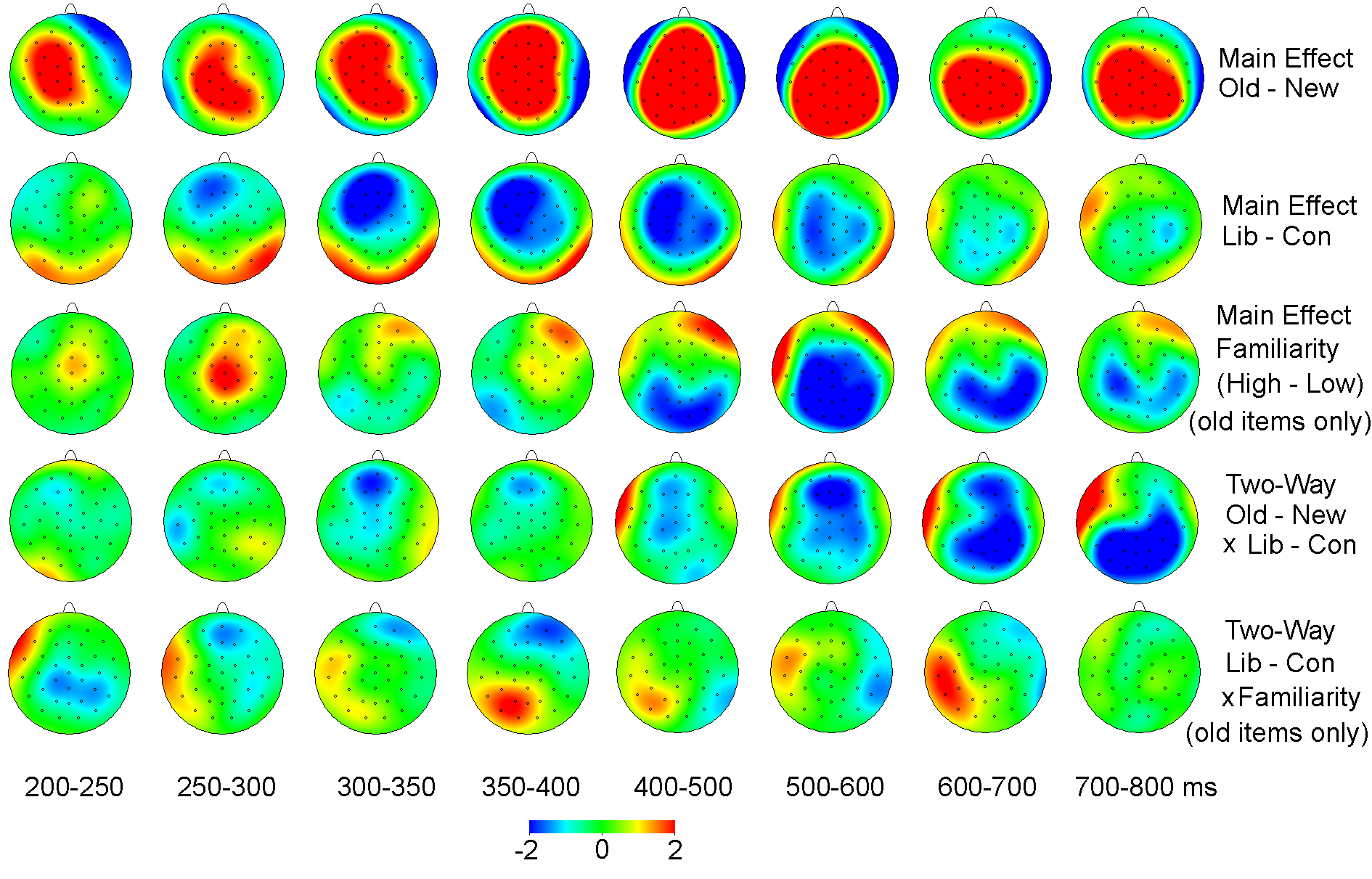

Supplement: Figure S2 — Statistical difference maps (t-values) of ERPs recorded in Experiment 2 for comparisons of old versus new items (Old/New), liberal versus conservative criterion (Lib/Con), highly familiar old items versus lowly familiar old items (Familiarity Old Items), and the two-way interactions of these differences. Mean t-values of the sample-by-sample t-test were calculated for the time-windows specified at the bottom. For a better illustration of the significant effects, the scaling was set to −2/+2 (corresponding to a t-value of approximately p = .05, uncorrected for multiple testing). (TIF) [file pone.0106411.s002.tif]

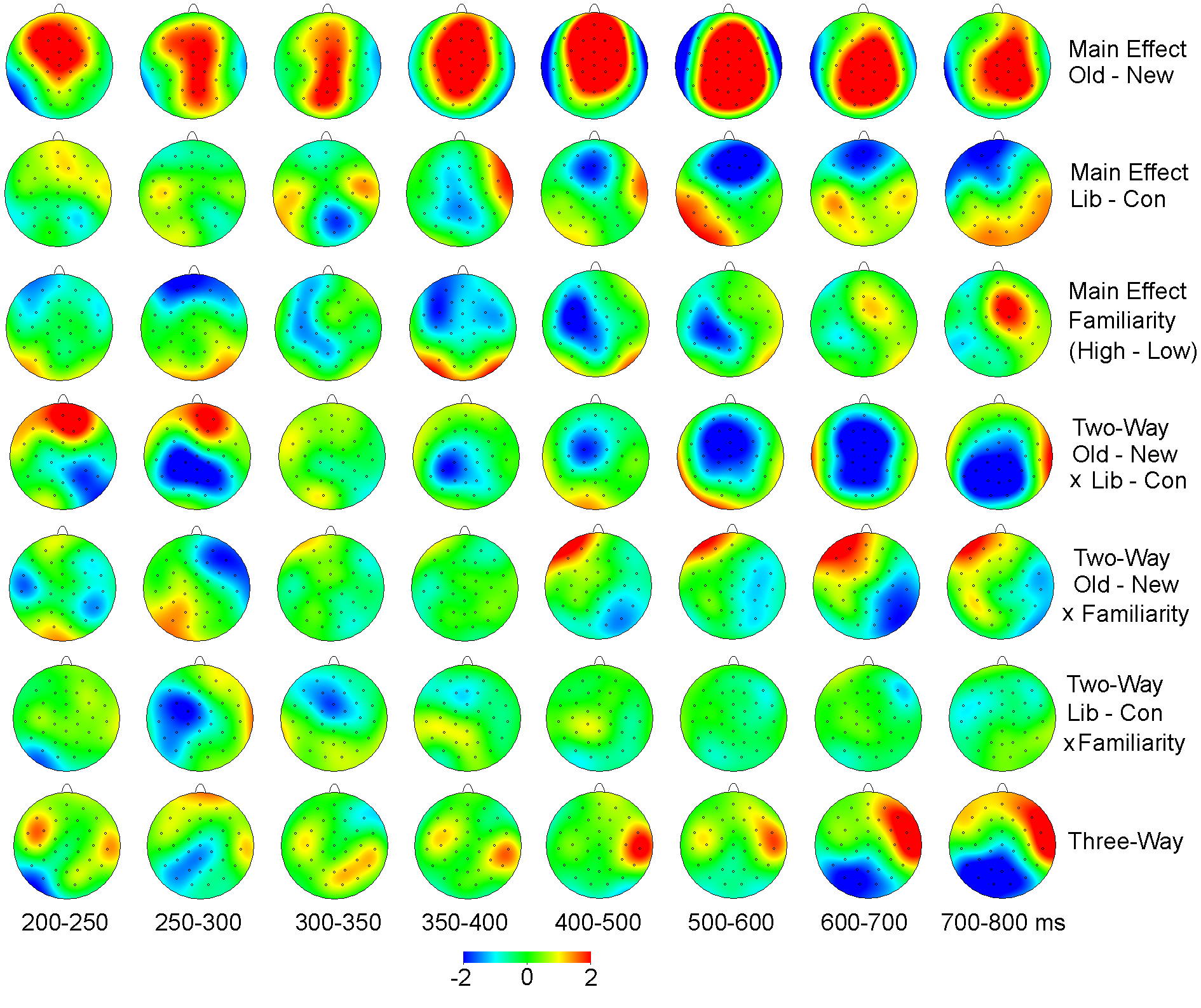

Supplement: Figure S3 — Statistical difference maps (t-values) of ERPs recorded in Experiment 3 for comparisons of old versus new items (Old/New), liberal versus conservative criterion (Lib/Con), highly familiar items versus lowly familiar items (Familiarity), and the two- and three way interactions of these differences. Mean t-values of the sample-by-sample t-Test were calculated for the time-windows specified at the bottom. For a better illustration of the significant effects the scaling was set to −2/+2 (corresponding to a t-value of approximately p = .05, uncorrected for multiple testing). (TIF) [file pone.0106411.s003.tif]

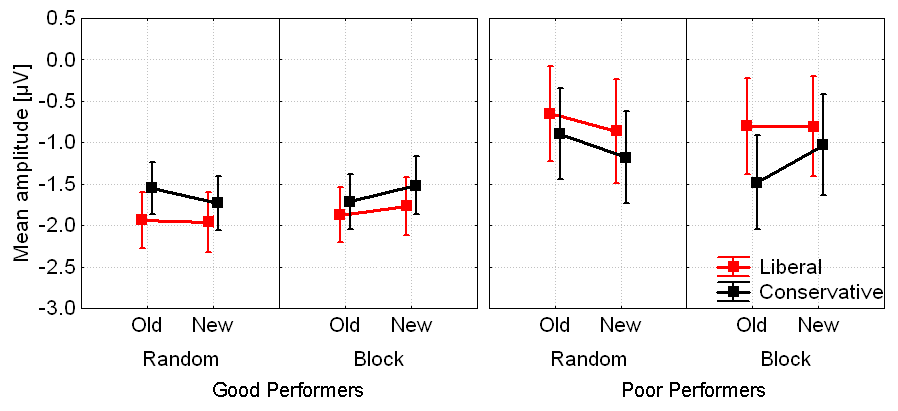

Supplement: Figure S4 — Mean ERP amplitudes (time-window 300–350 ms poststimulus) of the frontocentral negativity (FN320) in Experiment 1 shown separately for N = 24 participants who varied their decision criterion in accordance with instructions (good performers) and N = 8 subjects who did not comply with the instructions (poor performers) in at least one of the experimental conditions. An ANOVA with the between-subjects factor Group and the repeated measures factors Electrode Site, Block (block/random), Criterion (liberal/conservative), and Old/New revealed the following significant effects: First, an interaction of Old/New x Block: F(1, 30) = 9.15, p = 0.0051, eta2 = 0.23 yielding old > new differences in the random condition that were reversed in the block condition, and secondly, an interaction of Group x Criterion: F(1, 30) = 20.68, p<0.0001, eta2 = 0.41, indicating a larger FN320 bias effect (liberal more negative than conservative) for the good performers that was reversed in the group of poor performers. (TIF) [file pone.0106411.s004.tif]
