# Supplementary material for: Examining Event-Related Potential (ERP) Correlates of Decision Bias in Recognition Memory Judgments
Source: PLoS One. 2014 Sep 29;9(9):e106411. doi: 10.1371/journal.pone.0106411 (PMC4180069; doi:10.1371/journal.pone.0106411)
Supplement: Table S1 — Pearson's correlations of behavioral indices of Two-High-Threshold Model with indices of parametric and nonparametric Signal Detection Theory (SDT). (DOCX) [file pone.0106411.s005.docx]

| Nonparametric SDT [4] | Experiment 1 | Experiment 2 | Experiment 3 |
| --- | --- | --- | --- |
| Br – b | .97, .97, .93, .94 | .91, .88, .94., .84 | .98, .99, .90, .97 |
| Pr – A | .94, .95, .83, .92 | .98, .98, .98, .97 | .95, .98, .92, .90 |
| Parametric SDT [2] |  |  |  |
| Br – c | .92, .91, .77, .72 | .98, .93, .996, .94 | .91, .95, .79, .92 |
| Pr – d’ | .89, .95, .80, .91 | .92, .90, .97, .93 | .97, .97, .91, .83 |

*Note*: Correlations are reported in the following order for the four experimental conditions: Experiment 1: liberal/random, liberal/block, conservative/random, conservative/block. Experiment 2 and 3: liberal/low familiarity, liberal/high_familiarity, conservative/low-familiarity, conservative/high_familiarity.
